# Biological Control of the Chagas Disease Vector *Triatoma infestans* with the Entomopathogenic Fungus *Beauveria bassiana* Combined with an Aggregation Cue: Field, Laboratory and Mathematical Modeling Assessment

**DOI:** 10.1371/journal.pntd.0003778

**Published:** 2015-05-13

**Authors:** Lucas Forlani, Nicolás Pedrini, Juan R. Girotti, Sergio J. Mijailovsky, Rubén M. Cardozo, Alberto G. Gentile, Carlos M. Hernández-Suárez, Jorge E. Rabinovich, M. Patricia Juárez

**Affiliations:** 1 Instituto de Investigaciones Bioquímicas de La Plata (INIBIOLP, CONICET, CCT-La Plata, UNLP), Facultad de Ciencias Médicas, La Plata, Argentina; 2 Instituto de Patología Experimental, Facultad de Ciencias de la Salud, Universidad Nacional de Salta, Salta, Argentina; 3 Coordinación de Gestión Epidemiológica, Ministerio de Salud Pública, Salta, Argentina; 4 Facultad de Ciencias, Universidad de Colima, Colima, México; 5 Centro de Estudios Parasitológicos y de Vectores (CEPAVE, CONICET, CCT-La Plata, UNLP), La Plata, Argentina; University of Perugia, ITALY

## Abstract

**Background:**

Current Chagas disease vector control strategies, based on chemical insecticide spraying, are growingly threatened by the emergence of pyrethroid-resistant *Triatoma infestans* populations in the Gran Chaco region of South America.

**Methodology and findings:**

We have already shown that the entomopathogenic fungus *Beauveria bassiana* has the ability to breach the insect cuticle and is effective both against pyrethroid-susceptible and pyrethroid-resistant *T*. *infestans*, in laboratory as well as field assays. It is also known that *T*. *infestans* cuticle lipids play a major role as contact aggregation pheromones. We estimated the effectiveness of pheromone-based infection boxes containing *B*. *bassiana* spores to kill indoor bugs, and its effect on the vector population dynamics. Laboratory assays were performed to estimate the effect of fungal infection on female reproductive parameters. The effect of insect exuviae as an aggregation signal in the performance of the infection boxes was estimated both in the laboratory and in the field. We developed a stage-specific matrix model of *T*. *infestans* to describe the fungal infection effects on insect population dynamics, and to analyze the performance of the biopesticide device in vector biological control.

**Conclusions:**

The pheromone-containing infective box is a promising new tool against indoor populations of this Chagas disease vector, with the number of boxes per house being the main driver of the reduction of the total domestic bug population. This ecologically safe approach is the first proven alternative to chemical insecticides in the control of *T*. *infestans*. The advantageous reduction in vector population by delayed-action fungal biopesticides in a contained environment is here shown supported by mathematical modeling.

## Author Summary

Triatomine bugs are the causative agent of Chagas disease (American trypanosomiasis), they transmit the parasite *Trypanosoma cruzi* by infectious blood-sucking. In the southern Cone of South America, *Triatoma infestans* is the major disease vector. The efficacy of chemical control strategies is mostly based on pyrethroid application; however, the growing reports of resistant bug populations in Argentina and Bolivia urges to find innovative vector control measures. Among alternative control tools, entomopathogenic fungi were already shown to be effective against *T*. *infestans*; furthermore, pyrethroid-resistant insects are unable to counter entomopathogenic fungal infection. Pheromone-based technologies are widely used to help improve the insecticide killing efficacy; here we will show the usefulness of the fungal biopesticide combined to *T*. *infestans* contact aggregation pheromone to reduce bug population. That this low cost, low-tech tool can help control *T*. *infestans*, regardless their susceptibility to current chemical insecticides, will be shown by field, laboratory and mathematical modeling assessment.

## Introduction

Chagas disease, caused by infection with the parasite *Trypanosoma cruzi*, is the most important vector-borne disease in Latin America. It currently affects about 7–8 M people [1]. The blood-sucking insect *Triatoma infestans* (Hemiptera, Reduviidae) is the most widespread and relevant vector of the disease in the southern region of South America. The major target of vector control programs are well-established domestic populations, although peridomestic and sylvatic habitats may also harbor populations of significant size. Residual spray with chemical insecticides has been the major vector control strategy, showing a significant success in reducing the transmission in many areas of the so called Southern Cone Initiative (Argentina, Bolivia, Brazil, Chile, Paraguay and Uruguay) launched in 1991 [2]. However, sustained financial and human resources are essential to decrease and maintain domestic populations to an acceptable low level in order to interrupt or reduce vectorial transmission of *T*. *cruzi*. In addition, after about 30 years of pyrethroid application, pyrethroid resistance foci are increasingly being documented in Argentina and Bolivia [3, 4].

Despite their recognized efficacy, the major drawback of using the traditional pyrethroid spraying is its limited effectiveness in certain regions, such as the Gran Chaco area [5]. In particular, insecticide efficacy depends not only on the mode and frequency of application and on the characteristics of the dwelling structures, but also on the potential development of insecticide resistance. Systematic and periodic insecticide application covering most sites and refuges within infested houses (difficult to achieve in inaccessible remote areas), together with house improvement (to reduce potential sites for house infestation) are required in order to achieve suppression of *T*. *infestans* [5, 6]. A reduction of the indoor population of triatomines is the best way to decrease the risk of human infection with *T*. *cruzi* [7].

Among alternative control methods, a box containing a formulation of the fungus *Beauveria bassiana*, has been tested in the laboratory and in the field [8]. The killing ability of this fungal formulation was similar against pyrethroid-susceptible (Py-S) and pyrethroid-resistant (Py-R) *T*. *infestans* strains [9]; furthermore, successive applications of *B*. *bassiana* were estimated to significantly reduce the risk of acquiring *T*. *cruzi* through an infective bite [9]. The horizontal transmission (auto-dissemination) of fungal conidia was estimated at different bug densities, showing to contribute significantly to the overall infection of the insect population [10]. Pheromone-based traps are of widespread and increasing use to detect, lure-and-kill, or disrupt mating, for many agronomy insect pests [11, 12]; most if not all of the attractants are volatile [13]. However, much less research related to such tools is available for the control of animal and human insect-borne diseases. Carbon dioxide is a well-known attractant of most blood-sucking arthropods including *T*. *infestans* [9, 14–16], though a major limitation is the cost, operation and efficacy of its use under field conditions [17].

Triatomine population growth and regulation depends on several environmental factors [18], the most relevant being temperature and humidity [19], habitat and refuge [20], and diet [21] as well as their own density, i.e., intra-specific competition [22, 23]. Several mathematical models of triatomine population dynamics have been developed, including some models that incorporate triatomine population control; however, most of them have focused on the effect of insecticide spraying [24, 25], with the exception of a mathematical model for biological control of *Rhodnius prolixus* using the parasitoid *Ooencyrtus trinidadensis* [26]. Stevens et al. [7] modeled the effect of intervention strategies, such as insecticide spray rate and spraying efficiency. In the absence of sylvatic populations, the frequency of insecticide application was predicted to have the largest influence on house infestation, and the vector’s population reproductive rate *R*_*0*_ to be most sensitive to the spraying rate. The possible impacts of environmental stochasticity on the evolution of vector-borne diseases have also been investigated mathematically [27].

Several evaluations of the potential of *B*. *bassiana* as an agent of biological control against triatomines have been carried out [9, 10, 28–30], as well as various biological aspects of the relationship between triatomines and *B*. *bassiana*, such as modes of action of the pathogen [31–33], the effects of molting and starvation on the susceptibility of *Rhodnius prolixus* to the pathogen [34], and the contribution of the horizontal transmission of conidia to the overall population infection events [9, 10]. It has also been shown that *B*. *bassiana* is compatible with deltamethrin and can be used combined with this chemical without being exposed to metabolic detrimental effects [35]. However, no model for predicting the dynamics of triatomine vector populations after exposure to this entomopathogenic species has been developed.

The purpose of this work was i) to evaluate reproductive and survival parameters of *T*. *infestans* after exposure to the entomopathogenic fungus *B*. *bassiana*, combined with signals attractive to the bugs in infective boxes, measured both in the laboratory and in field assays, and ii) to develop a mathematical model of the effect of *B*. *bassiana* on *T*. *infestans* populations based on parameter estimates of those laboratory and field results. This information will allow an evaluation of the potential effectiveness of *B*. *bassiana* infection on the population dynamics of *T*. *infestans*, and the possibility of reducing *T*. *infestans* domestic populations.

## Materials and Methods

### Insects

The *T*. *infestans* colony of pyrethroid-susceptible (Py-S) insects used for laboratory assays was reared at 30°C, 50–60% relative humidity, under a 12:12h photo cycle, and fed on chickens every seven days. The colony is renewed yearly by incorporating first generation insects, usually from Formosa province, provided by the Servicio Nacional de Chagas, Cordoba, Argentina. For the laboratory bioassays we used insects two weeks after molting and after 1 week of a blood meal, except otherwise specified in the text.

### Fungal strain and formulation

A commercial powder formulation (WP) of *B*. *bassiana* strain GHA provided by Laverlam International (Butte, MT, USA) containing 1.27×10^11^ conidia/g (98% viable) was formulated with diatomaceous earth (DE) (Perma-Guard Inc., Albuquerque, NM, USA) in a conidia:DE ratio (2:1 w:w). DE produced no mortality at this dose. The commercial fungus powder was suspended in distilled water containing 0.01% Tween 80 for insect susceptibility bioassays.

### Effect of fungal infection on female fecundity and oviposition

Virgin females and males (15–20d old) were used 2–3d after blood-feeding on chickens and paired at random; each pair was placed on separate containers and checked for copulation completion following established protocols in our laboratory [36]; non copulated females were discarded. Twenty-eight females were randomly selected and allowed to be in contact with the fungal powder (2.6 × 10^8^ conidia/cm^2^) for 5 minutes [10]. Another set of 18 randomly selected females were used as controls. All females (infected and non-infected) were maintained in separate containers at 28°C and 60%RH. Reproductive parameters (oviposition, fecundity and fertility) were recorded daily until death in infected females and for eight weeks in control females. Oviposition refers to the number of females laying eggs per total females; fecundity refers to the number of eggs laid per female per day, and the fertility refers to the percentage of the laid eggs that hatched.

### Effect of exuviae on the aggregation behavior of *T*. *infestans*

Fifth instar *T*. *infestans* exuviae were washed with distilled water, left to dry on filter paper and ground with mortar and pestle until obtaining a fine powder that was then mixed with distilled water (0.038 g/ml). For bioactivity assays, this suspension was applied with a brush on half of the bottom’s internal surface of a 15×12 cm cardboard box (called the test box) and let to dry. Four replicates were set and, to completely randomize the assay, the location of the painted zone was rotated in each replicate 90° clockwise. The test box had two rectangular holes of 2.5×0.6 cm in two opposing sides at ground level to allow for the entry of insects; the test box was placed in the center of a larger container (60×60×50 cm) with the upper side covered with muslin cloth. Twenty-five 5th instar nymphs were released at random in the bottom of the larger container. Four and 24 h after the start of the experiment, the two boxes were opened and a picture was taken; the insects inside the exuviae-painted and the exuviae-free boxes were counted.

### Laboratory assay combining *T*. *infestans* exuviae suspensions and *B*. *bassiana* powder formulation

To evaluate the performance of an attraction-infection device, we used similar boxes treated as described above, and the conidia:DE formulation (2:1 w:w) powder was added over the same surface (100 mg/box). Twenty-five 5th instar non-infected nymphs were released in the container. Twenty four hours later, the boxes were opened and the insects collected, and then maintained individually at rearing conditions. Bug mortality was checked daily; dead insects were kept in individual humid chambers to confirm fungal infection [9]. Another set of infection assays were performed employing similar boxes without the exuviae suspension (controls). Three replicates of the treatment and of control assays were carried out. Median lethal time (MLT) was estimated as Σ (days_n_ × dead insects_n_)/total dead insects.

### Field box design

**Fig 1:**
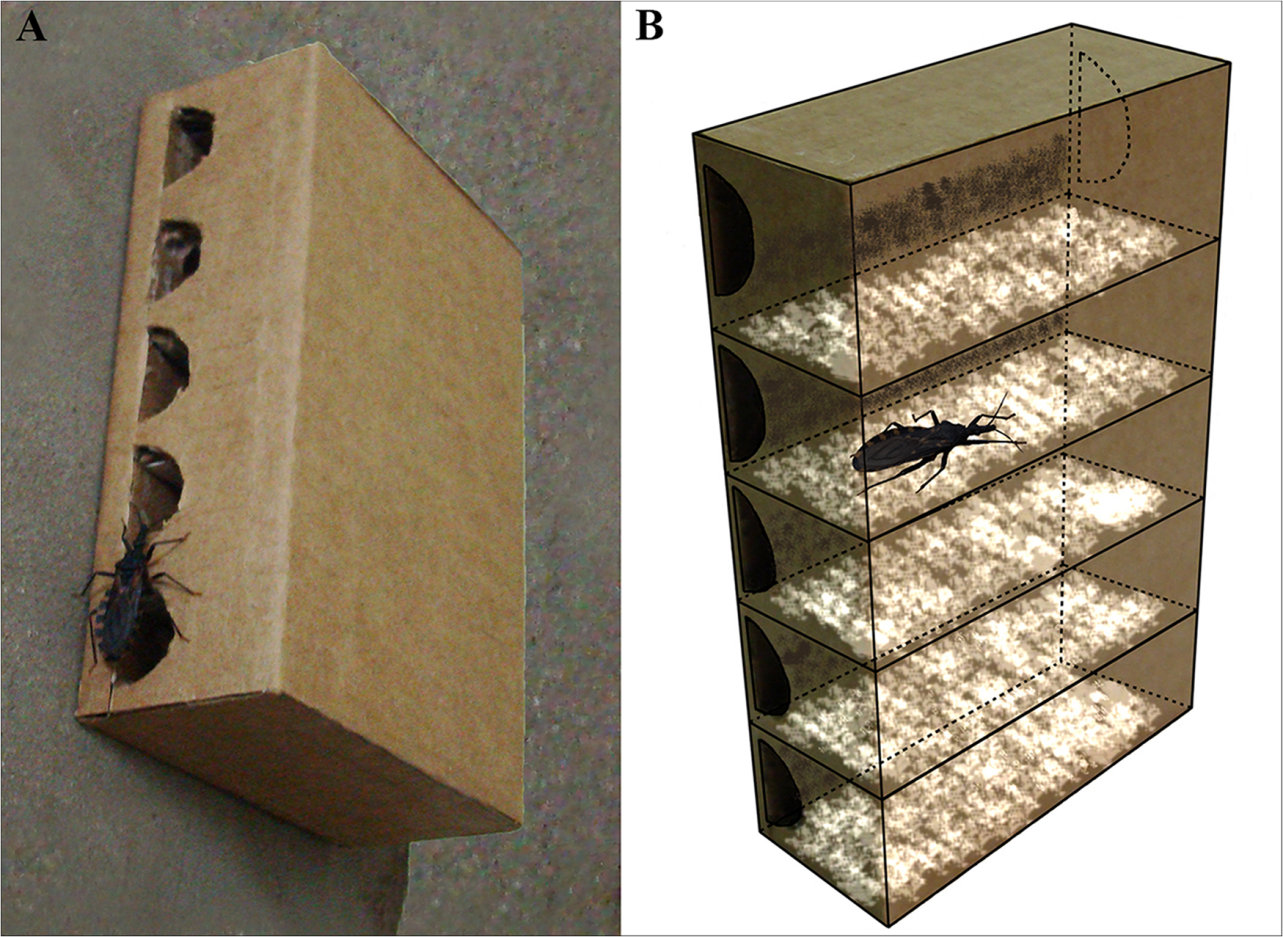
Field box. A: Picture showing a fifth instar nymph entering into the box. B: Scheme showing the inner box design; the upper side of the racks and the internal sides of the box were covered by the exuviae suspension (brown); the fungal formulation (white) was applied over the exuviae-painted rack surfaces.

The device consisted in a 3-mm medium density fiberboard (MDF) box of 16×11×4 cm, with five lateral holes placed at the same level than that of five internal racks (3 cm width) containing the conidia:DE mixture (2:1) (A scheme is shown in Fig 1). Before incorporating the fungal mix, the upper side of the racks and the internal sides of the boxes were covered with the exuviae suspension.

### Field assay

The field box was tested in rural houses of the Salvador Mazza Department, Salta Province, Argentina (22° 03’ S, 63° 41’ W; 804 masl), previously reported heavily infested with pyrethroid-resistant (Py-R) *T*. *infestans* [9]. A total of 13 houses were selected based on reports of vector presence. The number of insects recorded by visual observation (see below) varied from 10 to 63 per house. The selected houses were mostly made of adobe; indoor walls were partially plastered, with zinc roofs and rustic cement floors. Households ranged from two to eleven people per house, in 1–2 bedrooms; dogs were reported to sleep outside. Peri-domiciliary structures were chicken coops. The experiment was started in November 2009. Prior to the assays, a two-person team searched by sight and counted the triatomines in room areas, household goods, and beds (one man-hour/house); no flushing out treatment was applied in order to avoid physical damage or affecting the behavior of the bugs. Six boxes per room containing each 1g of fungal formulation (conidia:DE mix, 2:1 w:w) were placed in each house wall, close to the insect shelters. One month later (December 2009), a 3-person team searched thoroughly for dead insects inside the boxes and their surroundings. Dead bugs were collected and stored separately in labeled containers and transported to the laboratory to verify fungal infection; the infection boxes were replaced by new ones. One month later (January 2010), the boxes were removed; and all insects detected (dead and alive) were collected using the flushing-out method with 0.2% tetramethrin (Icona, Buenos Aires, Argentina) as an irritant agent, and transported to the laboratory in labeled flasks. Mortality was checked until 40 days after the boxes were removed, and cadavers were analyzed to verify fungal infection.

### Statistical analysis

The differences between the mean values obtained in the experiments described in 1.3, 1.4, and 1.5 were determined by the Student’s *t*-test (P < 0.05). Instat 3.05 (GraphPad Software Inc., San Diego, CA) was used for all statistical analyses.

### The mathematical model and estimation of the model’s parameters

We developed a stage-specific matrix model based on the seven stages of *T*. *infestans*: the egg, the five nymphal instars, and the adult stage; the model is a female-only model; the nymphal stages cannot be differentiated by sex, but no sex-ratio correction was needed because the sex-ratio of eggs after being laid is 1:1, and there is no sex-bias in the susceptibility to the fungal pathogen. The time unit of the model was one day.

**Fig 2:**
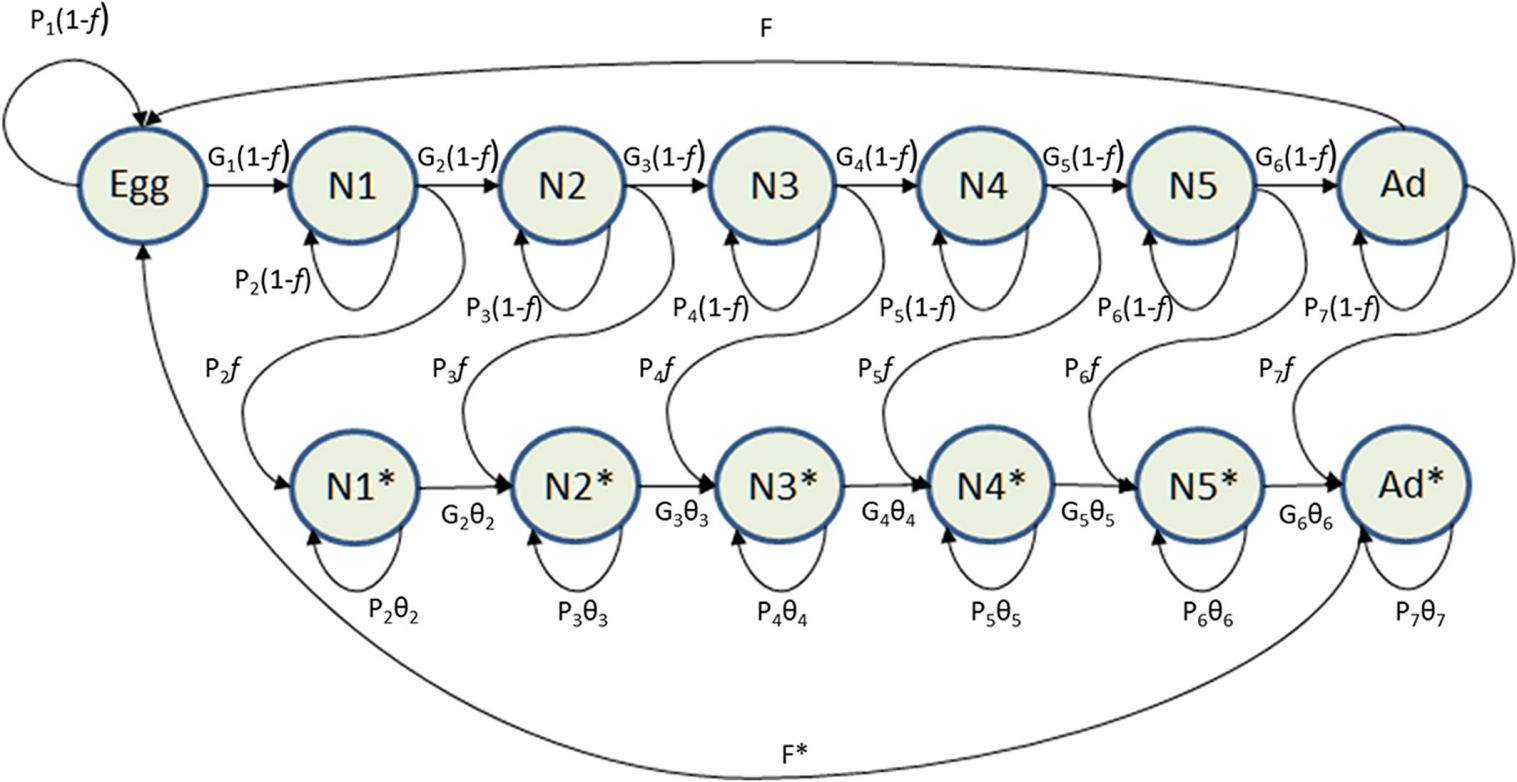
Life cycle diagram of the *T*. *infestans* matrix population model. Asterisks identify infected individuals. N1—N5 refers to nymph stage 1 through stage 5 of *T*. *infestans*. Parameters and symbols are defined in the text.

As our interest was to model the infection process of *T*. *infestans* by *B*. *bassiana* we added to this basic matrix model the infected individuals (with only six stage-specific classes, because eggs do not get infected by *B*. *bassiana*). Fig 2 shows a graphical representation of the matrix model in terms of a life cycle diagram, where the asterisks identify infected individuals; the upper row shows the non-infected insects with their probability of surviving within the same stage given by *G*_*i*_ (the “self-loop” arrows),while the probability of surviving within the same stage and molting to the next stage is given by *P*_*i*_ (the straight-line arrows); here *i* identifies the stage; *f* represents the force of attraction of all boxes to the vector population per time unit, so that for non-infected individuals *P*_*i*_ and *G*_*i*_ are multiplied by (1-*f*).

The lower row shows the infected insects with *P*_*i*_ and *G*_*i*_ as before, where *θ*_*i*_ (0≤ *θ*_*i*_ ≤ 1) is a measure of the mortality induced by the pathogen *B*. *bassiana*; that is, if the probability that a non-infected individual does not die before the next time unit is *P*_*i*_ +*G*_*i*_, then for an infected individual that probability is reduced by the factor *θ*_*i*_, that is it becomes (*P*_*i*_ +*G*_*i*_) *θ*_*i*_. The so-called “transitions” *P*_*i*_ *f* (the “curly” arrows) refer to the probability that an individual will survive in the same stage and get infected at the next time unit (it will “pass” from *N*_*i*_ to *N*_i_*). In this model, we ignore the probability that an individual will acquire the infection and molt to the next stage in the same time unit (that is, in the same day).

The egg stage has three entering arrows: eggs that were laid by non-infected and infected bugs, and eggs that having been laid in a given time unit, are surviving to the next time unit; however, the exit from the egg stage is only one arrow: from the egg to the first non-infected instar (an egg can only become a non-infected *N*_*1*_, with a “transition” given by *G*_*1*_(1-*f*)). The fecundity components are *F* and F*, for non-infected and infected bugs, respectively, both in units of female eggs/female/day (the long curved arrows pointing to the eggs at the top and at the bottom of the diagram). The corresponding algebraic expression of this life cycle diagram is given in S1 File, which also provides an example of a numerical matrix.

#### Estimation of the force of attraction of all boxes *(f)*

To calculate the force of attraction of all boxes (*f*) we have to estimate *c*_*n*_, the probability that an average triatomine individual would be attracted per unit time to any of the *n* boxes available; for that purpose we define *r* as the probability that an average susceptible individual will be infected by an infected individual per unit time (i.e., *r* represents horizontal transmission outside the infection box), so that

$$f=c_{n}*\left(1-r\right)+\left(1-c_{n}\right)*r$$ (1)

This equation is the sum of two terms: the left term (*c*_n_*(1-*r*)) is the probability that an individual will be attracted by a box and not infected by an infected individual, and the right term ((1-*c*_*n*_)* *r*) is the probability that an individual will not be attracted by a box and infected by an infected individual. That is, the daily rate of infection with in the boxes is given by the first term and the horizontal transmission (outside the boxes) by the second term. The estimation of *c*_*n*_ and *r* is described below.

If each box has a probability *α* of attracting an individual from the bug population per unit time, the probability that one bug is not being attracted is (1 - *α*) per unit time, so the probability of not being attracted by *n* boxes per unit time is (1 - *α*)^*n*^; consequently the probability of an individual from the bug population of being attracted by at least one box in *d* time units is given by:

$$c_{n}=1-{\left(1-\alpha \right)}^{\left(n*d\right)}$$ (2)

Eq 2 can be used to solve for α if we know the fraction of bugs attracted by the boxes (*c*_*n*_), how many boxes were set-up (*n*), and for how long the boxes had been exposed (*d*).

#### Estimation of *P*_*i*_ y *G*_*i*_

The probability of surviving in the same stage or graduating alive to the next stage (*P*_*i*_ and *G*_*i*_, respectively) is calculated by:

$$P_{i}=1-{\left(mean\;time\;in\;stage\;i\right)}^{-1}$$ (3)

$$G_{i}=\left(1-P_{i}\right)*P\left(Survival\;to\;stage\;i+1\right)$$ (4)

The data to estimate the mean time in stage *i* and the survival from stage *i* to stage *i*+1 was obtained from a variety of bibliographic sources (see Table A in S2 File).

#### Estimation of the horizontal transmission rate, *r*

To estimate the daily probability of horizontal transmission per (infected) bug (*r*), we used the results of an experimental house trial [10], where *I*_*o*_ is the number of initial infected bugs, *x* is the number of bugs that became infected from *S* initially susceptible bugs after a period of *T* days. So if *x*/*S* is the proportion of susceptible individuals that became infected by horizontal transmission then *R* ^*E*^_*o*_ (average number of infective contacts caused by one infected individual) is given by

$$R^{E}{}_{o}=-\frac{\left(I_{0}+S\right)\ln\left(1-w\right)}{I_{0}+x}$$ (5)

*R* ^*E*^_*o*_ can be considered as being composed by the product of two quantities: (a) the contact rate *λ*, and (b) the average time that an infected individual remains infective 1/μ, i.e., *R* ^*E*^_*o*_ = *λ*/*μ*. However, if each individual has a probability *p* of becoming infected in each contact, then *R* ^*E*^_*o*_ becomes *λp/μ*. As 1/*μ* can be obtained experimentally, and *R* ^*E*^_*o*_ is estimated from Eq 5, the estimate of the horizontal transmission rate *r* (the probability that an individual becomes infected on a given day) can be obtained by defining the probability that an average individual bug does not become infected as exp(-*λpw*), thus the probability that an average individual bug becomes infected is *r* = 1—exp(-*λpw*).

#### Estimation of *θ*_*i*_, the mortality induced by the pathogen

To estimate the risk of death of infected individuals we used the Hazard Ratio (HR), defined as the relative risk of death of infected individuals as compared to the death risk of non-infected ones. The HR is time invariant (it does not change from day to day), and it is estimated by the “Cox regression”. For this procedure we used the data from Forlani et al [10], pooling the data in three categories: [N1,N2,N3], [N4,N5], and [Adult]. The detailed survival analysis of each of these three categories, using the Kaplan-Meier estimation method of estimation can be found in S2 File.

An assumption behind the proportional hazard model is that the hazard in one group is a constant proportion of the hazard in the other group; i.e., the hazard ratio is a relative measure of the effect and tells us nothing about absolute risk; the estimate of the hazard ratio is formed by dividing the hazard rate under “treatment” (exposure to the pathogen in this case) by the hazard rate of the controls; thus the expression for the HR may be given as the probability that an individual infected by the pathogen in stage *i* will die in a given day, divided by the probability that an individual not infected by the pathogen in that same stage *i* will die in a given day; we have already provided an estimate of those probabilities (Eqs 3 and 4), so the stage-specific hazard rate (*HR*_*i*_) can be expressed as:

$$HR_{i}=\frac{1-\left(G_{i}+P_{i}\right)\theta_{i}}{1-\left(G_{i}+P_{i}\right)}$$ (6)

Now we can solve for *θ*_*i*_, which results in:

$$\theta_{i}=\frac{1+HR_{i}\left(G_{i}+P_{i}-1\right)}{\left(G_{i}+P_{i}\right)}$$ (7)

#### Estimation of the effects of the fungal pathogen on fecundity

For the infected bugs we resorted to the results of the experiment described in 1.3, and for the fecundity of non-infected individuals we used experimental data from the literature, where the *T*. *infestans* bugs were fed upon a variety of food sources and fed at different intervals, and kept under various temperature and humidity conditions; this variety of conditions provides fecundity estimates closer to those of the domestic field conditions. A table with the values of *T*. *infestans* fecundity used, their corresponding feeding and environmental conditions, and the bibliographic sources are given in Table B in S2 File.

#### Evaluation of the effect of the fungal pathogen on the population growth rate of *T*. *infestans*

After having estimated all parameters, and given the population matrix model, we selected the net population reproductive rate (*R* ^*P*^_*o*_) as indicator of the performance of the fungal pathogen’s effect on the triatomine population dynamics (the superscript *P* in *R* ^*P*^_*o*_ is used to differentiate it from *R* ^*E*^_*o*_, the epidemiological infection propagation). The parameter *R* ^*P*^_*o*_ describes the mean number of offspring by which a newborn individual will be replaced by the end of its life [37]. When *R* ^*P*^_*o*_ = 1 the population remains stable, while when *R* ^*P*^_*o*_<1 and when *R* ^*P*^_*o*_>1, the population decreases or increases, respectively.

In a stage-specific model, like in our matrix population model, *R* ^*P*^_*o*_ is defined as the dominant root of **R**, where **R** is a matrix defined as the product of **FN**, **F** being a matrix with the expected number of offspring produced per time step, and **N** another matrix, called the fundamental matrix, that provides the expected number of time steps spent in each transient stage [37]. To estimate *R* ^*P*^_*o*_ from the matrix model we used the software MATLAB [38], and for final simulations of the change of *T*. *infestans* population as a function of time we used the software R [39].

#### The “Efficacy” factor

The probability *α* (box attraction of individuals from the bug population per unit time) was estimated from assays in experimental houses described in Pedrini *et al*. [9], and as the box efficacy’s under field conditions, the parameter *α* was affected by a factor we called “*Efficacy*” (*E*). This factor was assigned a series of low arbitrary values (between 0.01 and 0.14) which were then used for multiplying *α*; doing this we were extremely conservative by assuming that the infection boxes in the field would have much lower efficacy than that obtained under laboratory conditions.

#### Sensitivity analysis of the model’s output to parameter values

There are four parameters more related to the impact of the fungal pathogen that have more uncertainties, either because they have been estimated for the first time: α (the attraction of boxes), and *R* ^*p*^_*o*_ (the bug’s population net growth rate), or because they have an arbitrary value (i.e., n: number of boxes, and *E*, the Efficacy factor). We selected Sobol’s method [40], based on a covariance analysis, for the estimation of the sensitivity of any output variable to all parameters, allowing for an interaction among parameters (Sobol’s *T* index); this index provides an estimation of absolute relevance among parameters. The sampling on the parameter space was the Winding Stairs sampling methodology [41], with 200 uniform random values for each parameter, resulting in 800 combinations. As output we selected two variables: the population net reproductive rate (*R* ^*p*^_*o*_), and the total triatomine population size at the end of an arbitrary simulation time period of 120 days.

### Testing the model with field data

Some of the main differences between the model and the field conditions are summarized in Table A in S3 File. In order to adapt field data and the model we converted the sampled house number of bugs of our field data in total number of bugs based on published relationships between the one man-hour/house collection and total house bug population (Table B in S3 File). Also, the pooled nymph field population estimates were separated into nymph stages based on published bug stage distribution (Table C in S3 File). To compare the model´s results with the field data we had to estimate some of the model parameters (Efficacy, α and N), and also the values of two new parameters called “Development time factor” and “Survival factor”, to correct those two variables from laboratory to field conditions (Table D in S3 File). For that purpose the model was then processed assigning to each of the five parameters a possible value selected from a uniform random distribution (within established lower and upper limits) trying to minimize the cumulative sums of squares function (SSQ) between the simulated and observed values of the four variables of interest: infected and non-infected nymphs and adults, respectively (Table E in S3 File). The model was used to simulate the house conditions for 60 days (the time frame of the field assays), with the field initial conditions and the best parameter estimates; 100 simulations were ran to obtain results for 100 combinations for each parameter, and the simulation results and the field data were compared with boxplots, for each of the four variables of interest (number of nymphs and adults infected and non-infected).

### Ethics statement

This study was performed within the guidelines established by the Bioethic Regulations of the Public Health Ministry of the Province of Salta, Argentina. Before the study, the objectives and the experimental protocol were explained during meetings with the community leaders, health professional and technical staff. Each head of family of these illiterate populations provided oral consent to perform the assay, after receiving the same information in the presence of local Vector Control Program officers, who participated as mediators. Oral consent was documented by a spreadsheet.

All animal care and laboratory experimental protocols were approved by theDirective Board of the INIBIOLP and carried out following the AVMA Animal Welfare Policies (https://www.avma.org/kb/policies/pages/default.aspx) and AVMA Guidelines on Euthanasia (https://www.avma.org/KB/Policies/Documents/euthanasia.pdf) (Instituto de Investigaciones Bioquímicas de La Plata’s Animal Welfare Assurance No. A5647–01).

## Results

### Laboratory assays

#### Exuviae suspension effect

**Fig 3:**
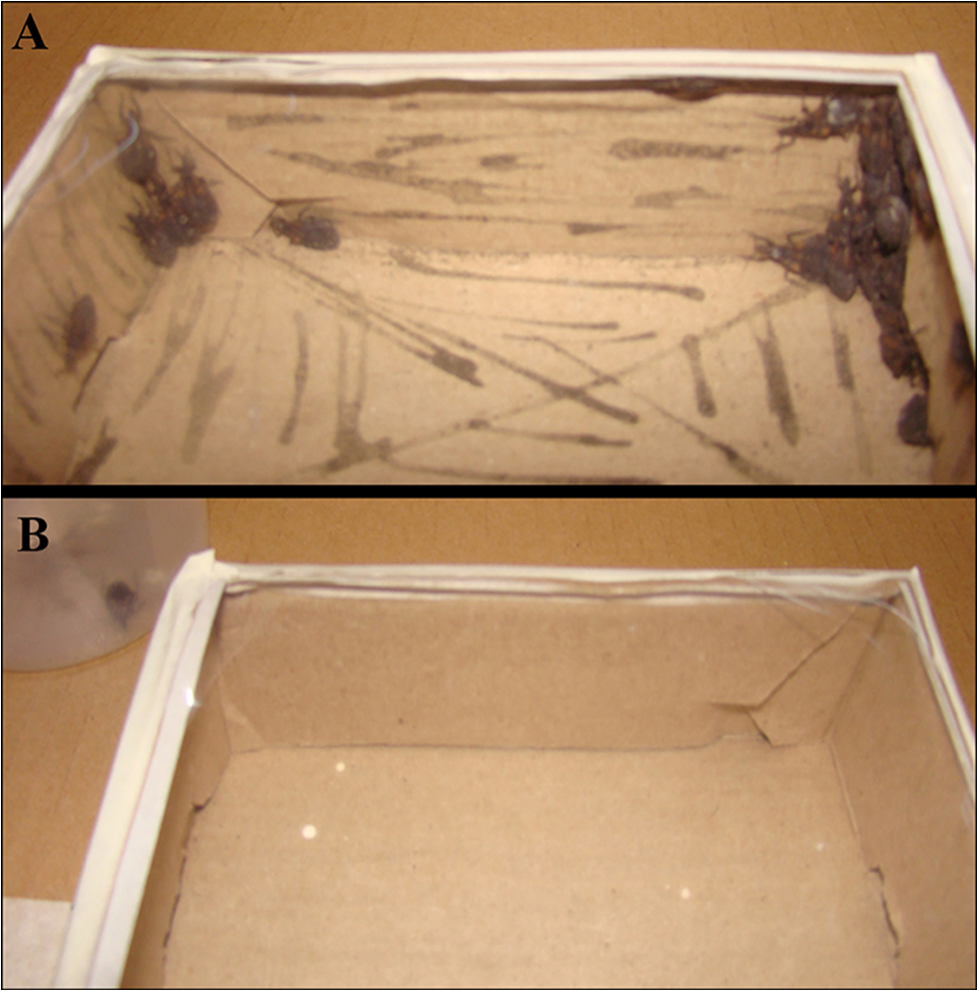
Aggregation response of *T*. *infestans* fifth instar nymphs exposed to an exuviae suspension. The arena was a square box half-painted with the exuviae suspension. A: painted surface, B: unpainted surface.

In this assay, 50 ± 4% of the insects entered the box after 4h, and 42.5 ± 16.4% of them were detected on the exuviae-painted surface. This percentage increased to 67 ± 6% after 24h, with 87.9 ± 10.2% of those bugs resting on the exuviae-painted surface; the difference between 4 and 24 h was statistically significant (*t* = 4.701, *P* = 0.0033) (Fig 3).

#### Assays of fungal formulation combined with insect exuviae

Bug mortality after 24h of exposure in boxes containing both fungi and exuviae was 59.0 ± 9.6%, while in the boxes containing the same fungal dose but no exuviae suspension, the bug mortality was significantly lower (33.3 ± 5.3%, t = 4.059, *P* = 0.0154). Corresponding MLT values were 8.9 ± 0.5 and 10.5 ± 2.2 days, respectively (*t* = 1.228, P = 0.2866) (Table 1).

**Table 1:** Mortality of *Triatoma infestans* exuviae combined with a fungal formulation on 5th instar nymphs.

Assay	Fungi	Exuviae	Mortality (% ± SD)
1	yes	no	33.3 ± 5.3*
2	yes	yes	59.0 ± 9.6*

* The differences between with and without exuviae were statistically significant (*p* < 0.05) by Student’s *t* test. There were 3 replicates for each assay, with 25 bugs per replicate.

#### Effect of fungal infection on female reproductive parameters

All the females in the control group laid eggs, but the oviposition percentage was reduced to about 50% in the infected group (Table 2). The total individual reproductive output of the uninfected (control) females was 35.6 ± 5.3 eggs/female/life, in an average period of 52 days, while the reproductive output of the infected females was 9.9 ± 2.3 eggs/female/life in an average life time period of 7.3 days; the difference in total number of egg per female per life between infected and non-infected females was statistically significant (t = 5.028, *p* < 0.0001). No significant difference was detected between the egg fertility in eggs laid by infected and control females (about 80% of all eggs hatched in both the treatment and the control) (Table 2). The fecundity of both infected and non-infected females was 0.99 ± 0.23 and 0.68 ± 0.10 eggs/♀/day, respectively; this difference was not statistically significant (*t* = 1.008, *p* = 0.3190). This lack of statistical significance was strongly biased by the much shorter than average longevity of our experimental observation period of the bug control population, and for this reason we resorted to estimate the average daily fecundity of *T*. *infestans* from the bibliography for modeling purposes (see Table B in S2 File).

**Table 2:** Effect of fungal infection on *Triatoma infestans* female reproductive parameters.

Parameter	Control	Fungus-treated
Oviposition (%)	100	57
Number of eggs laid / ♀ / life (mean ± SEM)*	35.6 ± 5.3	9.9 ± 2.3
Fecundity (eggs / ♀ / day) (mean ± SEM)	0.68 ± 0.10	0.99 ± 0.23
Fertility (% ± SEM)	85.9 ± 2.1	84.0 ± 4.0
Number of females	18	28

* The difference between the fungus-treated and the control experiments was statistically significant (*p* < 0.0001) by Student’s *t* test.

#### Field assays

Total insects recorded at the start of the experiment and two months after the installation of the infection boxes in field houses are shown in Table 3, together with the total number of insects found dead throughout the experiment. Before the intervention, 145 nymphs and 86 adults were detected, and at the end of the experiment, a total of 223 nymphs and 89 adults alive were counted. Of the insects collected, a total of 53 nymphs and 76 adults resulted dead by fungal infection in the experimental period. The percentage of dead insects /house during the assay period corresponded to 20.1 ± 5.1% nymphs and 52.1 ± 7.5% adults; with a mean mortality percentage of 37.2 ± 6.2% for the nymph and adult pooled population. Insects dead by fungi include insects that were already dead at the collection time together with those insects caught alive (but already infected), and that died during a 40 day-evaluation period, as described in Materials and Methods. Adult mortality was estimated as 81.9 ± 12.9% and nymph mortality was 45.0 ± 14.7% when compared to the initial number of bugs detected, the combined population mortality (pooling nymphs and adults) was 62.2 ± 10.7%.

**Table 3:** *Triatoma infestans* mortality results of the field assays in rural houses.

House^a^	November 2009		January 2010					
	Number of insects counted		Number of live insects detected		Number of insects dead by fungi		Percentage of insects dead by fungi^b^	
	Nymphs	Adults	Nymphs	Adults	Nymphs	Adults	Nymphs	Adults
1	22	11	76	27	17	21	18.3	43.7
2	19	0	0	3	0	5	-	62.5
3	29	34	59	14	8	4	11.9	22.2
4	17	9	26	14	18	15	40.9	51.7
5	3	7	16	7	3	7	15.8	50.0
6	8	13	25	20	3	8	10.7	28.6
7	11	10	16	2	1	10	5.9	83.3
8	36	2	5	2	3	6	37.5	75.0

^a^ At the end of the experiment, data was collected in 8 out of the 13 houses initially selected, because some of them were dismantled and others reconditioned.

^b^ Calculated as the number of insects dead by fungi/number of total insects detected (dead and alive) at the end of the experiment × 100. Insects counted as dead correspond to those already dead at the collection time and insects caught alive (but already infected), that died during the 40 day-evaluation period.

### Modeling results

#### Survival parameters

The estimation of the mean time in a given stage and the stage-specific survival parameters, based on Eqs (3) and (4), are shown in Table 4.

**Table 4:** Stage-specific mean development time (average time in stage, in days), and stage-specific probability of survival to the next stage of *Triatoma infestans*; *G*_*i*_ and *P*_*i*_ were calculated with Eqs 3 and 4, respectively.

Stage (*i*)	Name	Mean time in stage	P (Survival to next stage)	*P*_*i*_	*G*_*i*_
1	Egg	19.0	0.8313	0.9474	0.0437
2	N1	20.0	0.8676	0.9499	0.0435
3	N2	24.2	0.8803	0.9586	0.0364
4	N3	30.3	0.8765	0.9669	0.0290
5	N4	40.6	0.8790	0.9754	0.0217
6	N5	63.9	0.8526	0.9843	0.0133
7	Ad	241.8	0	0.9959	0.0000

N1—N5 refers to nymphs stage 1 through stage 5.

The data of the mean time in stage *i* and the survival from stage *i* to stage *i*+1 was obtained from several of bibliographic sources (see Table A in S2 File).

#### Force of attraction of boxes

From the results of Pedrini et al. [9] 58% of bugs were attracted by six boxes in a 4-days period, so applying Eq (2) we obtained an estimate of *α* = 0.0355, by solving for α from: 0.58 = 1- (1- α)^(6*4).

#### Horizontal transmission

The results of the auto-dissemination of *B*. *bassiana* conidia estimated by Forlani et al. [10] indicated that under experimental house conditions, of 629 susceptible bugs exposed to 90 infected bugs, 267 individuals were infected in a 25 day period, thus μ = 1/25 and *x* = *I*_*0*_/*S* = 362/629 = 0.5755. Applying this value to Eq (5) we obtained an estimate of *R* ^*E*^_*o*_ of 1.11271, and *λp* = *R* ^*E*^_*o*_μ = 1.11271/25 = 0.0445. Assuming that every bug entering a box with the fungus pathogen becomes infected, then *x* is equivalent to *c*_*n*_ (the probability that an average triatomine individual would be attracted per unit time to any of the *n* boxes left per house); the estimated value of the horizontal transmission rate (*r*) will thus vary depending upon the number of boxes (*n*) and the efficacy factor (*E*), but remains constant along the simulation time if *n* and *E* are constant.

#### *T*. *infestans* stage-specific pathogen induced mortality (*θ*_*i*_)

The results of the estimation of HR for the different bug categories used ([N1,N2,N3], [N4,N5], and [Adult]) are given in Table 5.

**Table 5:** Estimation of the stage-specific hazard ratios (HR) of the effect of *Beauveria bassiana* on *Triatoma infestans* and their 95% confidence intervals based on the stage-specific mortality rate, pooling stages into three categories.

Stage category	Mortality (%)	HR	Lower 95% CI	Upper 95% CI
N1-N3	93.8	97.12	93.59	100.65
N4-N5	97.9	57.80	55.04	60.55
Adult	83.3	25.00	20.77	29.23

N1—N5 refers to nymphs stage 1 through stage 5.

When we applied the stage-specific *G*_*i*_ and *P*_*i*_ surviving probability values of Table 4 to Eq 6 (using the same *HR*_*i*_ value for those stages in the same category), we obtained the stage-specific estimates for *θ*_*i*_ given in Table 6.

**Table 6:** Stage-specific estimates of the mortality factor due to the effect of the fungal pathogen (*θ*_*i*_).

Stage	HR	*θ*_*i*_
Egg	1	1.0000
N1	97.12	0.3584
N2	97.12	0.5216
N3	97.12	0.6061
N4	57.8	0.8303
N5	57.8	0.8687
Adult	25	0.9003

N1—N5 refers to nymphs stage 1 through stage 5.

#### Effectiveness of the fungal pathogen

**Fig 4:**
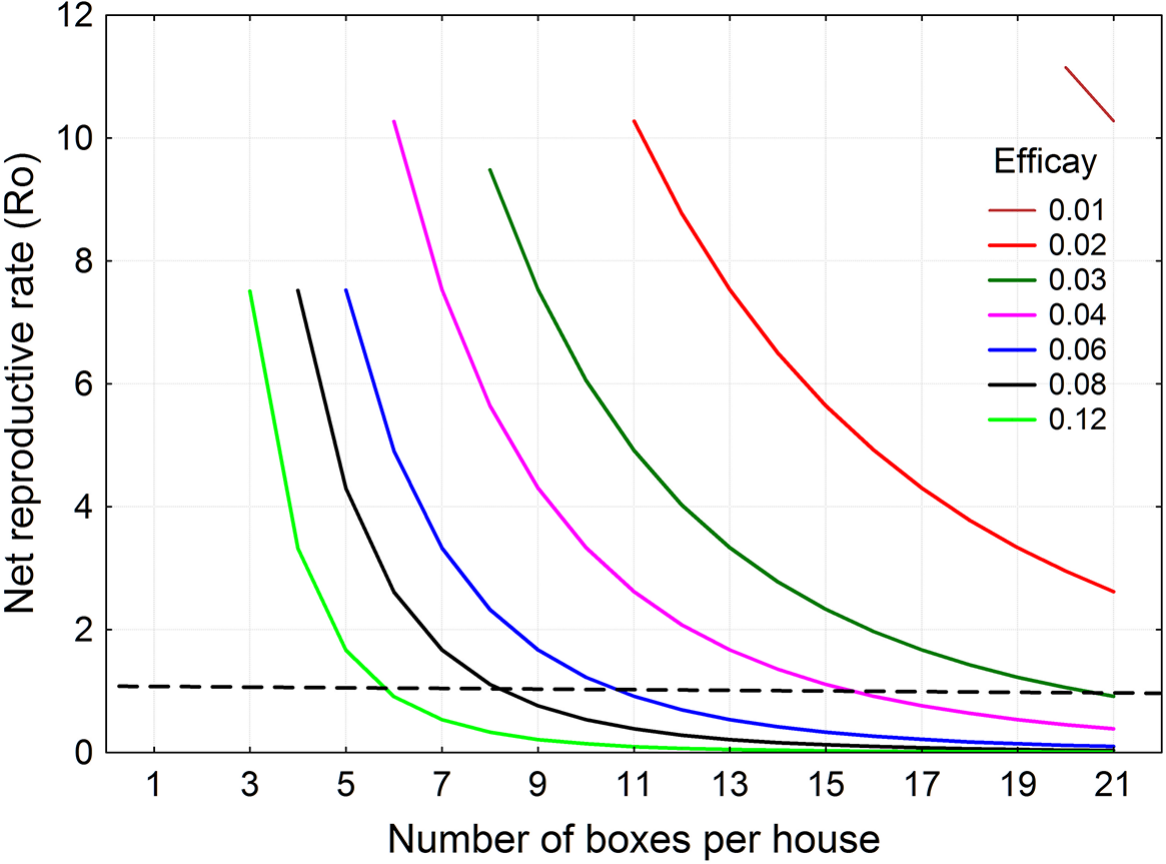
Performance of the fungal pathogen on the triatomine population dynamics based on the net population reproductive rate (*R*_*0*_), as a function of the box Efficacy factor and the number of boxes/house. The black hatched horizontal line indicates the value of *R*_***0***_ = 1, above which the triatomine population always increases; below that line the triatomine population always decreases.

**Fig 5:**
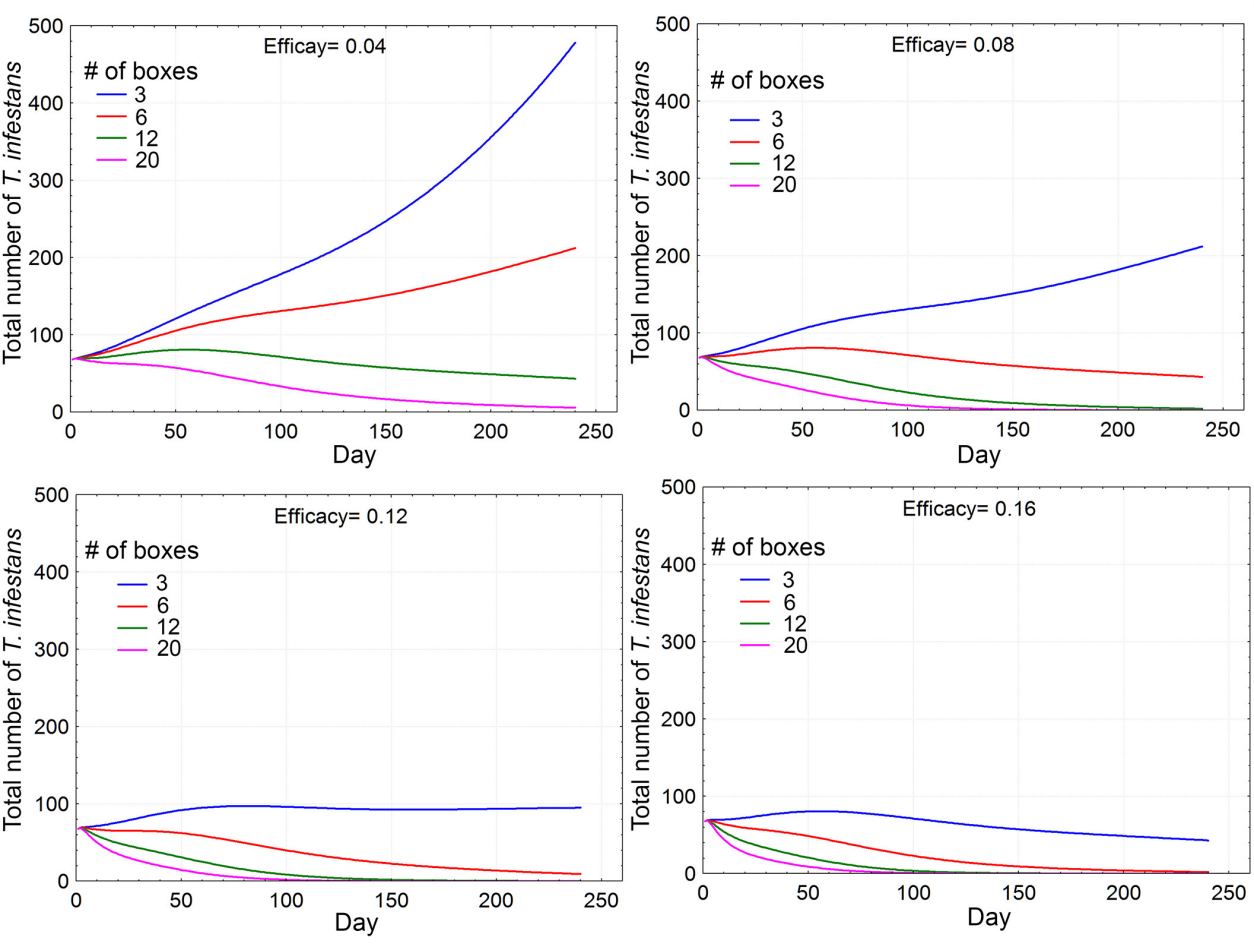
Combination of the Efficacy factor (*E*) and the number of boxes per house (*n*) to maintain the net population growth rate (*RoP*) within a band of ±10% around 1.

The bug’s net population reproductive rate (*R* ^*P*^_*o*_), will depend not only on the number of boxes used but also on the efficacy (*E*) of the boxes under field domestic conditions. As the latter is not known we ran several simulations of the model under different combination of values of these two parameters (efficacy factor and number of boxes used); the results of a selection of those combinations are shown in Fig 4 with the bug population of one simulated house for eight months. We see that for efficacy (*E*> 0.10) as few as 4–5 boxes per house would be enough for a progressive reduction of the bug population *R* ^*P*^_*o*_<1; however, with lower efficacy (0.05 <*E*< 0.1) more than 8–10 boxes would be needed. Fig 5 shows how different combinations of the number of boxes and the efficacy provide the same value of *R* ^*P*^_*o*_≈ 1. Interestingly, assuming no infection boxes were present, the value of *R* ^*P*^_*o*_ is 87, close to the ones published in the literature (see bibliographic sources in Table C in S2 File).

**Fig 6:**
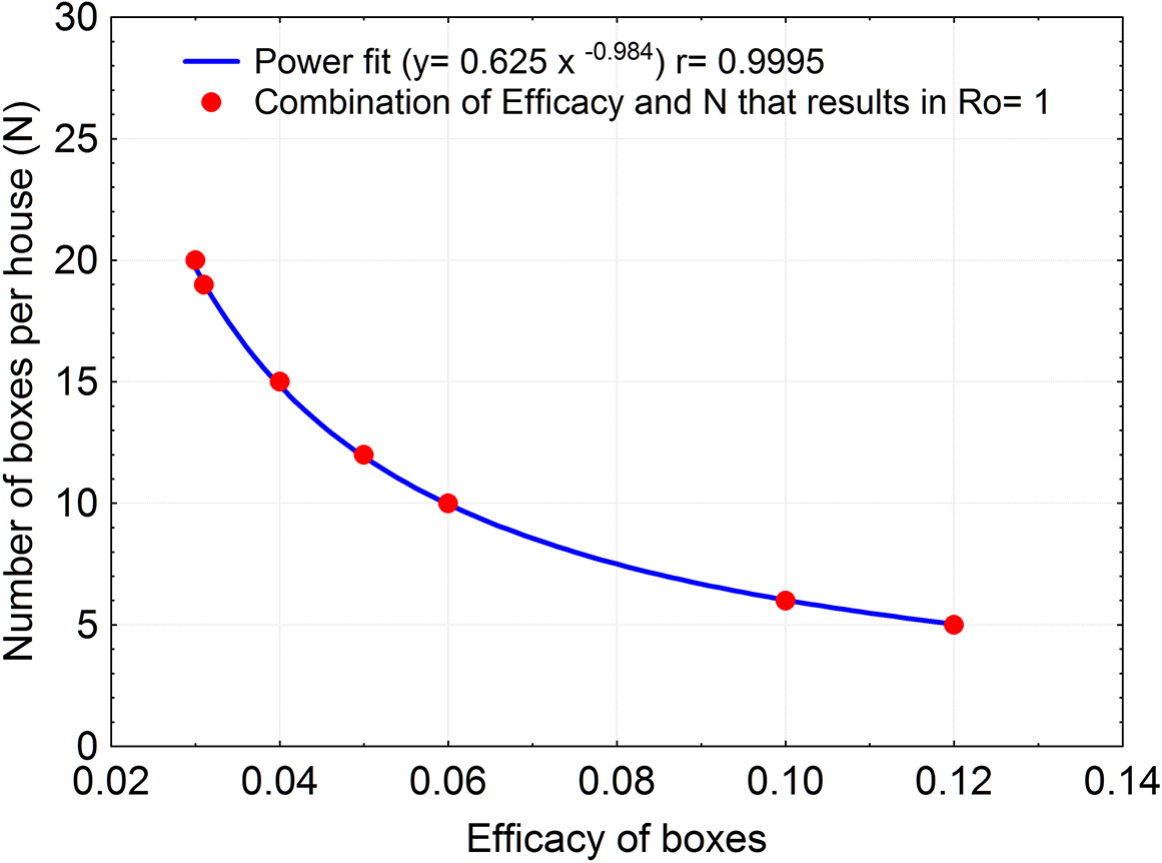
Simulated time series of the effects of the pathogen on the size of a *T*. *infestans* population during 240 days for four selected values of the box Efficacy factor (*E*), and four selected number of boxes (*n*) per house.

Fig 6 shows the progressive effects of *B*. *bassiana* on the size of a *T*. *infestans* population during 8 months (240 daily simulation time steps) for several values of the number of boxes per house and four selected values of the efficacy factor (*E*). Even at low *E* the triatomine population size is always reduced, more quickly (i.e., earlier in time) as the number of boxes increases. At *E* >0.08, any number of boxes above 3 results in a reduction of the bug population.

#### Sensitivity analysis of the model

**Fig 7:**
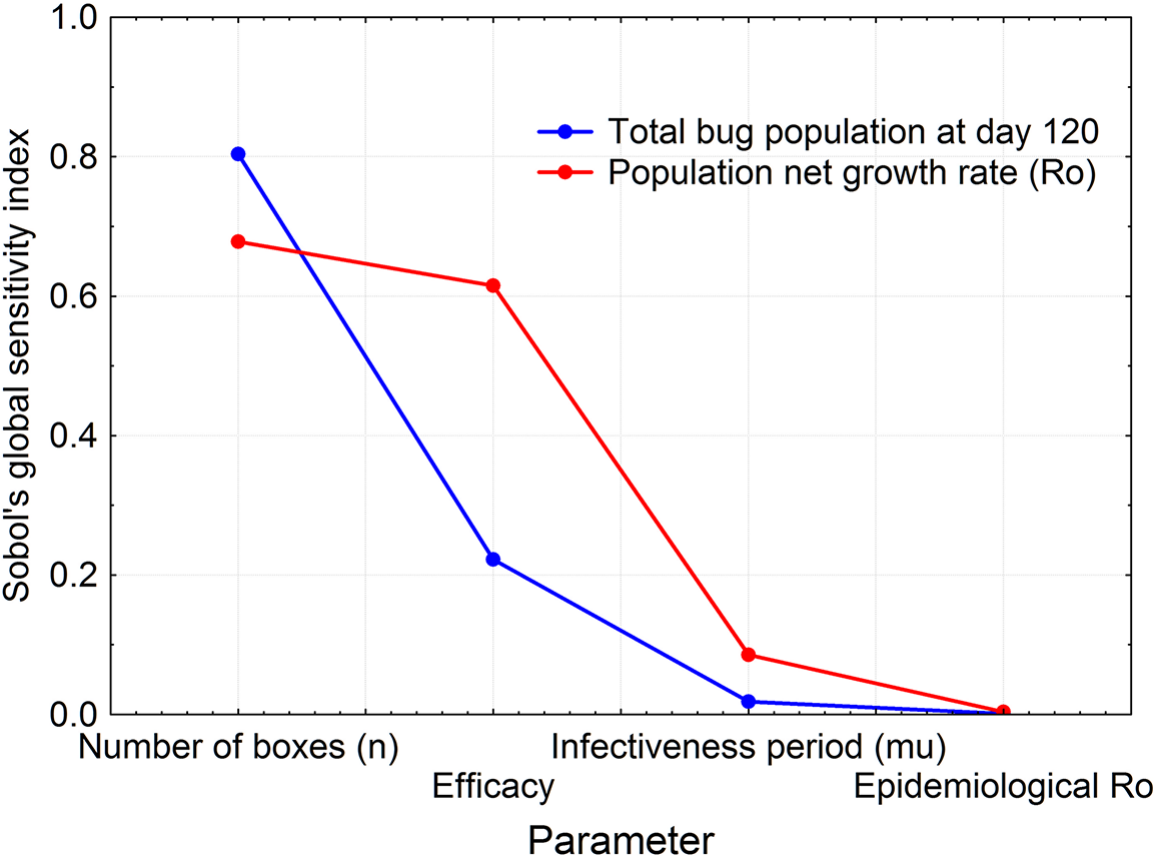
Sobol’s *T* index of parameter importance of four of the model’s parameters in their effects on two model output variables: the net rate of population increase (*R*_*o*_), and triatomine population size at day 120 of the simulations.

Fig 7 shows the result of Sobol’s method assessing the relative importance of four parameters to two output variables: the population net reproductive rate (*R* ^*P*^_*o*_), and the total triatomine population size (at time 120 days). Both output variables showed a very similar response to the changes in the four parameters selected for sensitivity, as expected because of the close relationship between both output variables. Among the four parameters tested, the number of boxes had the highest order of importance followed by the efficacy (*E*).

#### Field data and model comparison

**Fig 8:**
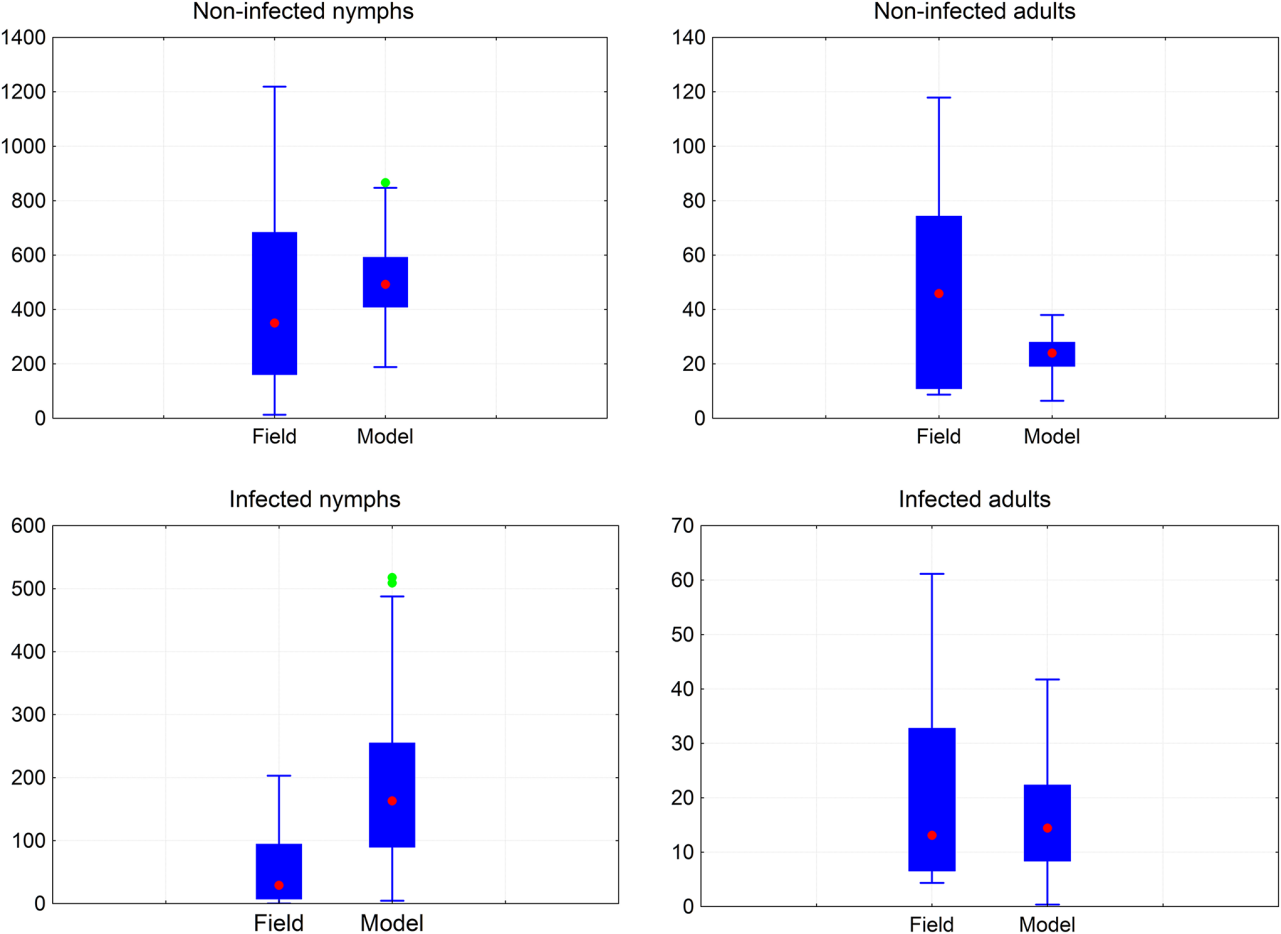
Boxplots of the model's predictions and the field data for infected and non-infected nymphs and adults. The blue boxes include 25%-75% of the values; the red dot is the median of the distribution of all values, the vertical bars identify the non-outlier range, and the green dots identify outliers.

The best parameter estimation values (Table E in S3 File) obtained were: Efficacy = 0.058, Fecundity = 0.56, N = 2, Development time factor = 0.85 and Daily survival factor = 0.82. Fig 8 compares the model’s predicted results with the field data. With one exception (the infected nymphs) the 95% confidence intervals around the mean of the model embrace the mean of the field values and *vice versa*; indicating that the house field data could be reproduced satisfactorily with the model.

## Discussion

### Attraction cues

Triatomine aggregation behavior is relevant to the survival and reproduction of *T*. *infestans* (and therefore to its population fitness) because it facilitates finding daylight shelters to evade predators, thus remaining motionless (and safe) for most of the day. The epicuticle lipids of *T*. *infestans* are involved in this behavior; among them, the free fatty acid fraction was identified as partially responsible for the aggregation response [8, 42]. Other cuticle components also elicit mating behavior in adult males upon contact [36]. However, none of these components have been tested in field assays. To take advantage of these aggregation properties of the epicuticle, and to enhance the attraction effect of the *B*. *bassiana* infection boxes, we used an exuviae suspension, with a lipid composition similar to that of the juvenile cuticle [43] which resulted in a significant aggregation response of *T*. *infestans* to conspecific cuticle-like signals [8]; the aggregation response persisted at least up to one month. The incorporation of this aggregation cue increased the efficacy of the bioinsecticide-containing box in a relatively short time frame; our present results show that bug mortality was significantly higher (59.0 ± 9.6%) when the fungus was combined with the aggregation cue, compared with 33.3 ± 5.3% mortality when the infection box contained only the fungus. We are not aware of similar attempts combining an entomopathogenic fungi and a non-volatile attractant cue in other vector control strategies. In the typical attract-kill paradigm extensively used for some agronomy pests [11, 12], the attractant is usually a low molecular weight volatile semiochemical that exerts its action at a certain distance [13]; we have not found any evidence on the use of relatively high molecular weight, short-range attractant compounds. Among potentially useful volatile signals, the limitations of using carbon dioxide under field conditions have been mentioned already mentioned [9, 17]. The chemistry of volatile defensive signals emitted by triatomines has been known for many years [44, 45]; they have been used for bug collection and infestation monitoring in long term field experiments (up to 6 months), showing a superior performance than the traditional man-hour survey collections [46]. However, no evidences are available on their efficacy in short term evaluation assays.

### Reproductive parameters

*T*. *infestans* populations have marked fluctuations under certain conditions, with about two population cycles per year, at least in the central region of Argentina [47], with fecundity as one of the main parameters affecting population size and also impinging on its population stability. In our results not only the percent of infected ovipositing females was significantly lower (57%) as compared to controls, but also total lifetime offspring production after fungal infection was reduced in about 70% of the corresponding controls, mainly due to the shortened longevity of adult infected females. When 18 complete cohort studies of *T*. *infestans* taken from different bibliographic sources were used, we found similar results: the average fecundity of un-infected females was 0.86 ♀eggs/♀/day, about 70% higher than for the infected females (0.51 ♀eggs/♀/day) (see Table B in S2 File).

### Field

Both *T*. *infestans* adults and nymphs are similarly susceptible to the entomopathogen *B*. *bassiana* [9, 10]; however our summer field assays showed that the adult population mortality (52.1 ± 7.5%) was higher than that of the juvenile stages (20.1 ± 5.1%). Sixty days after starting the assay, the mean percentage mortality was estimated as the total number of dead insects/house compared to total number of insects detected (dead and alive)/house (Table 3). These results are probably related to seasonality; nymph’s populations show a marked annual peak during the hot season [47]; on the contrary, the same authors reported a scarce 2% fluctuation for the adult population during summer.

Similar previous trials in winter, when bug population is relatively stable and inactive [47], were performed at a neighboring rural locality distant about 30 km from the current experimental site, using the same fungal formulation (1 g per box) and the same number of boxes (6 boxes per room), showing 52.4% overall bug mortality one month after setting the boxes [9]. That is, after 30 days, bug mortality was similar to our present results, despite the 2008 assay was carried out in the winter season, and CO_2_ was used to attract the triatomines. A constrain in the experiments here described was the timing. It has already being addressed that the seasonal timing of vector control interventions can greatly affect its efficacy [48, 49]. Gorla and Schofield [47] proposed the cold season (June-August) as the most appropriate, because recovery of any surviving populations would be inhibited by low temperatures. However, operational difficulties forced us to perform our field assays in the warm season (late Spring to Summer).

### Biological control

In vector indoor control the most important advantages of the use of biopesticides over chemical insecticides are the smaller risk of developing host resistance, and minimal risk to the environment and other organisms [50]. In contrast to the well-known irritant effect of chemical insecticides, the powdered fungal formulation was not avoided, but rather showed some attractiveness to *T*. *infestans*. During the biopesticide experiments, insects exhibited a characteristic camouflage behavior: using their front legs to partially cover themselves with soil dust; and they did the same with the dusty powder formulation. The paradigm of “instant-kill requirement” of chemical insecticides can be contrasted to the “slow-kill” bioinsecticide effect, with the latter showing many advantages. Mycoinsecticides elicit no irritation response on the host, and by reducing the survival of the host without instant killing (i.e., slow-kill), allow for the fungus a long term control with lower possibilities of developing resistance [51]. This was shown by Read et al. [52], using a mathematical model of the use of mycoinsecticides with mosquitoes transmitting malaria. Also in relation to mosquitoes, Koella et al. [53] extended the idea of a late mode of action by biopesticides, and claimed that entomopathogenic fungi might act as an “resistance-evolution-proof” insecticide for mosquito control because of its relatively late action in the mosquitoes’ life-cycle, allowing the reproduction of part of the adult population before they are killed, thus preventing the evolution of resistance.

We have observed that fungal infection can affect triatomine feeding behavior; i.e., shortly after insects are infected with fungi (2–3 days), most of them are not apt to extend their proboscis to suck blood; this effect is similar to the one reported in infected mosquitoes [54]. As *T*. *cruzi* transmission is highly related to early diuresis after feeding, the poor feeding aptitude induced by *B*. *bassiana* not only reduces the fecundity (strongly dependent upon blood ingestion) but also the potential of disease transmission (lack of quantitative data prevented us to include this information in the model).

### Model

The mathematical model we developed is a relatively simple one, and we consider it as a first step in developing a more realistic model. E.g., this is a deterministic model, despite both demographic and environmental stochasticity (unpredictable spatio-temporal variability in environmental conditions and feeding sources, as well as variability in life history traits) are among the main factors of the fluctuations in the density of triatomine populations. Notwithstanding the strictly deterministic characteristics of the model here developed, and the various simplifications we resorted to, our sensitivity analysis results proved extremely useful; they tell us that the number of boxes (*n*) and the boxes’ efficacy factor (*E*), are the main drivers of the reduction of the total bug population, with the former being about four times as strong as the latter in their effects. This result implies that, being *E* an arbitrary factor to compensate for potentially overestimating the effect of the fungi from laboratory results, the performance of *B*. *bassiana* as a biological control agent should be quite reliable, and that whatever environmental condition that decreases the efficacy may be compensated by increasing the number of boxes. This is clearly seen in Fig 5, which shows how *E* and *n* are related to maintain the net population growth rate *R* ^*P*^_*o*_≈ 1. The extremely low “importance” of the infectiveness period (*μ*), *R* ^*P*^_*o*_ and *R* ^*E*^_*o*_ parameters in the behavior of the model, also suggest the structure and the demographic and epidemiological parameters are reasonable because *μ* and *R* ^*E*^_*o*_ subsume the essential parameters of the dynamics of infection: *μ* is related to the average time that an infected individual remains infective (1/*μ*), and *R* ^*E*^_*o*_ represents the average number of infective contacts caused by one infected individual; we can then conclude that even if our laboratory and field estimates of those parameters would have been flawed, their impact on the model’s predictions would be minimal. Additionally, it indicates that as the behavior of the model is more sensitive to external factors such as the efficacy (*E*) and the number of boxes (*n*) than to the biological parameters, we are confident on the prospects of our model´s predictions, because they depend essentially on factors that can be manipulated during the biological control campaign operations.

Furthermore, the population reduction obtained in the field assays after 2 month intervention, using 6 boxes per room, is similar to that predicted by the model (for 6 boxes/room) when *E* ~ 0.12, a conservative value for the box efficacy (Fig 6).

This figure shows that for this number of boxes, the condition *R*_*0*_< 1 can be attained with an extremely low efficacy (*E* = 0.06). Modifying the number of boxes, the model predicts that the population can still be decreased using 3 boxes, but with higher efficacy (*α* = 0.16); on the contrary, at extremely low efficacy (*E* = 0.02), 20 boxes would be needed for *R*_*0*_< 1. The good fit obtained in the comparison between model prediction and house field data (Fig 8) suggests that, despite the original model’s structure was not intended to simulate a real house, the model’s performance can be considered satisfactory. Nevertheless, to be more realistic future versions of the model should include bioclimatology, house construction materials, number of rooms in the house, number of people, number of animals, and peri-domiciliary structures, a task that was beyond our original purpose.

The future of chemical control of *T*. *infestans* based on indoor insecticide spraying as the sole tool for bug suppression is threatened by growing incidents of pyrethroid-resistant population detection, together with the known operational and financial difficulties to spray a large number of sparsely populated small villages located in remote areas. Thus, it is evident the urgent need to modify the conventional vector control methodologies, at least in the Gran Chaco area. Under this avenue, it is relevant to gain additional understanding of the effect of biological tools in vector population control-*per se* or jointly applied with chemicals-, and we think that our combined results of field, laboratory and mathematical modeling suggest that a formal pilot program of *T*. *infestans* population biological control with *B*. *bassiana* is in order.

## Supporting Information

S1 File Algebraic and numerical matrix representation of the life cycle diagram of Fig 2. (DOCX)

S2 File Kaplan-Meier survival analysis. (DOCX)

S3 File Field data and model comparison. (DOCX)
