# Supplementary material for: Biological Control of the Chagas Disease Vector Triatoma infestans with the Entomopathogenic Fungus Beauveria bassiana Combined with an Aggregation Cue: Field, Laboratory and Mathematical Modeling Assessment
Source: PLoS Negl Trop Dis. 2015 May 13;9(5):e0003778. doi: 10.1371/journal.pntd.0003778 (PMC4430541; doi:10.1371/journal.pntd.0003778)
Supplement: S1 File — (DOCX) [file pntd.0003778.s001.docx]

**S1 File**

***Algebraic and numerical matrix representation of the life cycle diagram of Fig.2.***

P_1_ 0 0 0 0 0 F 0 0 0 0 0 F*

G_1_P_2_*(1-f_2_) 0 0 0 0 0 0 0 0 0 0 0

0 G_2_*(1-f_2_) P_3_*(1-f_3_) 0 0 0 0 0 0 0 0 0 0

0 0 G_3_*(1-f_3_) P_4_*(1-f_4_) 0 0 0 0 0 0 0 0 0

0 0 0 G_4_*(1-f_4_) P_5_*(1-f_5_) 0 0 0 0 0 0 0 0

0 0 0 0 G_5_*(1-f_5_) P_6_*(1-f_6_) 0 0 0 0 0 0 0

0 0 0 0 0 G_6_*(1-f_6_) P_7_*(1-f_7_) 0 0 0 0 0 0

0 P_2_*f_2_ 0 0 0 0 0 P_2_****θ***_2_ 0 0 0 0 0

0 0 P_3_*f_3_ 0 0 0 0 G_2_****θ***_2_P_3_****θ ***_3_ 0 0 0 0

0 0 0 P_4_*f_4_ 0 0 0 0 G_3_****θ***_3_P_4_****θ***_4_ 0 0 0

0 0 0 0 P_5_*f_5_ 0 0 0 0 G_4_****θ***_4_P_5_****θ***_5_ 0 0

0 0 0 0 0 P_6_*f_6_ 0 0 0 0 G_5_****θ***_5_P_6_****θ***_6_ 0

0 0 0 0 0 0 P_7_*f_7_ 0 0 0 0 G_6_****θ***_6_P_7_****θ***_7_

| 0.9474 | 0 | 0 | 0 | 0 | 0 | 0.86 | 0 | 0 | 0 | 0 | 0 | 0.51 |
| --- | --- | --- | --- | --- | --- | --- | --- | --- | --- | --- | --- | --- |
| 0.0437 | 0.9293 | 0 | 0 | 0 | 0 | 0 | 0 | 0 | 0 | 0 | 0 | 0 |
| 0 | 0.0425 | 0.9378 | 0 | 0 | 0 | 0 | 0 | 0 | 0 | 0 | 0 | 0 |
| 0 | 0 | 0.0357 | 0.9460 | 0 | 0 | 0 | 0 | 0 | 0 | 0 | 0 | 0 |
| 0 | 0 | 0 | 0.0284 | 0.9542 | 0 | 0 | 0 | 0 | 0 | 0 | 0 | 0 |
| 0 | 0 | 0 | 0 | 0.0212 | 0.9630 | 0 | 0 | 0 | 0 | 0 | 0 | 0 |
| 0 | 0 | 0 | 0 | 0 | 0.0131 | 0.9743 | 0 | 0 | 0 | 0 | 0 | 0 |
| 0 | 0.0206 | 0 | 0 | 0 | 0 | 0 | 0.3404 | 0 | 0 | 0 | 0 | 0 |
| 0 | 0 | 0.0208 | 0 | 0 | 0 | 0 | 0.0156 | 0.5000 | 0 | 0 | 0 | 0 |
| 0 | 0 | 0 | 0.0210 | 0 | 0 | 0 | 0 | 0.0190 | 0.5861 | 0 | 0 | 0 |
| 0 | 0 | 0 | 0 | 0.0211 | 0 | 0 | 0 | 0 | 0.0176 | 0.8098 | 0 | 0 |
| 0 | 0 | 0 | 0 | 0 | 0.0213 | 0 | 0 | 0 | 0 | 0.0180 | 0.8551 | 0 |
| 0 | 0 | 0 | 0 | 0 | 0 | 0.0216 | 0 | 0 | 0 | 0 | 0.0116 | 0.8966 |

**Figure A.** Symbolic projection matrix for a *T. infestans* population with infected and non-infected individuals and the probability transitions among them. The asterisk represents the multiplication operation (except for F*, that represented the fecundity of infected females). Symbols and parameter identification are given in the main text.

**Figure B.** Numerical projection matrix for a *T. infestans* population with infected and non-infected individuals and the probability transitions among them. This matrix is shown only for the purpose of illustration, and corresponds to the symbolic matrix of Fig. 1 for the following parameter values: number of boxes = *n* = 1, box efficacy = 0.1, horizontal transmission rate = *r* = 0.01825, probability of one box attracting an individual per unit time = *c_n_*= α = 0.0355, force of attraction of all boxes = *f* = 0.02167.
