# Supplementary material for: Biological Control of the Chagas Disease Vector Triatoma infestans with the Entomopathogenic Fungus Beauveria bassiana Combined with an Aggregation Cue: Field, Laboratory and Mathematical Modeling Assessment
Source: PLoS Negl Trop Dis. 2015 May 13;9(5):e0003778. doi: 10.1371/journal.pntd.0003778 (PMC4430541; doi:10.1371/journal.pntd.0003778)
Supplement: S2 File — (DOCX) [file pntd.0003778.s002.docx]

**S2 File**

**Kaplan-Meier survival analysis**

1. Pool [ Nymph 1, Nymph 2, Nymph 3 ]

|  |  |  | Censored | |
| --- | --- | --- | --- | --- |
| Case | N | N° of events | N° | Percent |
| Control | 120 | 3 | 117 | 97.5% |
| Treated | 146 | 137 | 9 | 6.2% |
| Global | 266 | 140 | 126 | 47.4% |

Mean and Median of survival time

| Case | Mean^a^ | | | | Median | | | |
| --- | --- | --- | --- | --- | --- | --- | --- | --- |
|  |  |  | 95% Confidence interval | |  |  | 95% Confidence interval | |
|  | Estimate | Standard error | Lower | Upper | Estimate | Standard error | Lower | Upper |
| Control | 20.858 | .094 | 20.675 | 21.042 |  |  |  |  |
| Treated | 7.911 | .420 | 7.088 | 8.734 | 6.000 | .355 | 5.304 | 6.696 |
| Global | 13.752 | .461 | 12.849 | 14.655 | 14.000 |  |  |  |

^a^ If there is censoring the estimate is limited to the longest survival time

1. Pool [ Nymph 4, Nymph 5 ]

|  |  |  | Censored | |
| --- | --- | --- | --- | --- |
| Case | N | N° of events | N° | Percent |
| Control | 80 | 12 | 68 | 85.0% |
| Treated | 143 | 140 | 3 | 2.1% |
| Global | 223 | 152 | 71 | 31.8% |

Mean and Median of survival time

|  |  |  | 95% Confidence interval | |  |  | 95% Confidence interval | |
| --- | --- | --- | --- | --- | --- | --- | --- | --- |
|  | Estimate | Case | Mean^a^ | Median | Estimate | Standard error | Lower | Upper |
| Control | 19.800 | .389 | 19.038 | 20.562 |  |  |  |  |
| Treated | 8.650 | .271 | 8.119 | 9.181 | 8.000 | .238 | 7.534 | 8.466 |
| Global | 12.650 | .423 | 11.821 | 13.479 | 10.000 | .438 | 9.141 | 10.859 |

^a^ If there is censoring the estimate is limited to the longest survival time

1. Adults

|  |  |  | Censored | |
| --- | --- | --- | --- | --- |
| Case | N | N° of events | N° | Percent |
| Control | 20 | 9 | 11 | 55.0% |
| 1 | 18 | 15 | 3 | 16.7% |
| Global | 38 | 24 | 14 | 36.8% |

Mean and Median of survival time

| Case | Mean^a^ | | | | Median | | | |
| --- | --- | --- | --- | --- | --- | --- | --- | --- |
|  |  |  | 95% Confidence interval | |  |  | 95% Confidence interval | |
|  | Estimate | Standard error | Lower | Upper | Estimate | Standard error | Lower | Upper |
| Control | 18.950 | .779 | 17.422 | 20.478 |  |  |  |  |
| Treated | 12.833 | 1.190 | 10.502 | 15.165 | 11.000 | .849 | 9.337 | 12.663 |
| Global | 16.053 | .855 | 14.377 | 17.729 | 17.000 | 3.082 | 10.959 | 23.041 |

^a^ If there is censoring the estimate is limited to the longest survival time

**Table A.** Estimates of the mean development time of *T. infestans* from Nymph 1 to the emergence of the adults, the laboratory conditions used for its estimation and their data sources.

| Case # | Average  laboratory  temperature (^o^C) | Blood  source | Average  feeding  frequency (days) | Development time (days) | | | | | | |
| --- | --- | --- | --- | --- | --- | --- | --- | --- | --- | --- |
|  |  |  |  | Egg | NI | NII | NIII | NIV | NV | Data source |
| 1 | 30 | heparinized bird blood | 7 | NA | 14 | 22 | 35 | 66 | 80.3 | 1 |
| 2 | 27 | Pigeon | 17 | 19 | 13 | 19 | 30 | 39 | 51.4 | 2 |
| 3 | 19.5 | Pigeon | 17 | 19 | 19 | 24 | 36 | 43 | 57 | 3 |
| 4 | 24.3 | Chicken | 2.5 | NA | 25 | 25 | 33.5 | 43.6 | 82 | 3 |
| 5 | 26 | Chicken | 7 | 19.8 | 31.2 | 32.3 | 17.4 | 11.1 | 48.9 | 4 |

**Data sources:**

1. de Isola, E., D. Sánchez y V. Katzin. 1980. *Triatoma infestans*: Influencia de la Alimentación Artificial sobre su Ciclo de Vida. Medicina (Buenos Aires) 40(1): 207-212.

2. Carcavallo, R. U. and A. Martínez. 1972. Life Cycles of some Species of Triatoma (Hemiptera: Reduviidae). The Canadian Entomologist 104(5): 699-704.

3. Da Rocha Carvalheiro, J. 1974. Tabuas de Vida e Capacidade Inata de Aumento Numérico de uma Populacao de *Triatoma infestans* em Condicoes de Laboratorio. III - Oviposicoes e Evolucao dos Ovos. Revista do Centro Academico Rocha Lima e do Hospital das Clinicas da Faculdade de Medicina de Riberao Preto da Universidad de Sao Paulo 6(1-2): 1-10.

4. Rabinovich, J. E. 1972. Vital statistics of Triatominae (Hemiptera: Reduviidae) under laboratory conditions. I. *Triatoma infestans* Klug. Journal of Medical Entomology 9(4): 351-370.

**Table B.** Estimate of the mean fecundity of uninfected *T. infestans*, the laboratory conditions used, and their data sources.

| Case # | Average  laboratory  temperature (°C) | Blood  source | Average  feeding  frequency (days) | Average # of  ♀eggs/♀/day | Data  source |
| --- | --- | --- | --- | --- | --- |
| 1 | 24-28 | Chicken | 7 | 1.26 | 1 |
| 2 | 27 | NA | NA | 0.71 | 2 |
| 3 | 26 | Chicken | 7 | 3.42 | 3 |
| 4 | 26 | Pigeon | 15 | 0.54 | 4 |
| 5 | 26 | Guinea pig | 15 | 0.32 | 4 |
| 6 | NA | Guinea pig | 1/10 | 0.61 | 5 |
| 7 | 21.3 | NA | NA | 0.90 | 6 |
| 8 | 30 | Heparinized bird blood | 7 | 0.40 | 7 |
| 9 | 27 | Pigeon | 17 | 0.87 | 8 |
| 10 | NA | Chicken | NA | 1.30 | 9 |
| 11 | 22 | Chicken | 7 | 0.18 | 10 |
| 12 | 27 | Chicken | 7 | 0.16 | 10 |
| 13 | 35 | Chicken | 7 | 0.17 | 10 |
| 14 | 26 | Chicken | 7 | 0.25 | 11 |
| 15 | 24.3 | Chicken | 2.5 | 0.62 | 12 |
| 16 | Ambient | Chicken | 3.5 | 1.22 | 13 |
| 17 | 26.5 | Chicken | 3.5 | 1.54 | 13 |
| 18 | 34 | Chicken | 3.5 | 1.08 | 13 |
|  |  |  |  | **0.86** | **Average** |
|  |  |  |  | **0.77** | **Std.Dev.** |

**Data sources:**

1. Perlowagora-Szumlewicz, A. 1969. Estudos sobre a Biología do *T. infestans*, o Principal Vetor da Doenca de Chagas no Brasil. (Importancia de Algumas de Suas Características Biológicas no Planejamento de Esquemas de Combate a EsseVetor). Revista Brasileira de Malariologia e DoencasTropicais 21: 117-59.

2. Gadea, J. 1984. Influencia de la Densidad en la Fecundidad de *Triatoma infestans* (Klug, 1834) (Hemipera: Reduvidae). Chagas 1(4): 21-28.

3. Rabinovich, J. E. 1972. Vital statistics of Triatominae (Hemiptera: Reduviidae) under laboratory conditions. I. *Triatoma infestans* Klug. Journal of Medical Entomology 9(4): 351-370.

4. Nattero, J., G. Leonhard, C. S. Rodríguez and L. Crocco. 2011. Influence of the Quality and Quantity of Blood Ingested on Reproductive Parameters and Life-Span in *Triatoma infestans* (Klug). ActaTropica 119: 183–187.

5. Lwoff, M., and P. Nicolle. 1943. Recherchessur la nutrition des réduvidéshémophages.II. Besoinsalimentaires des adultes de *Triatoma infestans* Klug dans les conditions habituelles d'élevage fécondité des femelles. Bulletin de la Société de pathologie exotique 36: 110-24.

6. Borda, M. R. 1971. Algunos Nuevos Aspectos sobre Biología y Ecología de *Triatoma infestans* Klug, 1834 y su Enemigo Natural Telenomusfariai Lima, 1927. Breves Notas Referentes a Trypanosoma cruzi Chagas, 1909. I Congreso Latinoamericano de Entomología, Cuzco, Perú. Manuscrito.

7. de Isola, E., D. Sánchez y V. Katzin. 1980. *Triatoma infestans*: Influencia de la Alimentación Artificial sobre su Ciclo de Vida. Medicina (Buenos Aires) 40(1): 207-212.

8. Carcavallo, R. U. and A. Martínez. 1972. Life Cycles of some Species of Triatoma (Hemiptera: Reduviidae). The Canadian Entomologist 104(5): 699-704.

9. Oscherov, E. B., M. P. Damborsky, M. E. Bar and D. E. Gorla. 2004. Competition between vectors of Chagas disease, *Triatoma infestans* and *T. sordida*: effects on fecundity and mortality. Medical and Veterinary Entomology 18: 323-328.

10. Schofield, C. J. 1982. The role of blood intake in density regulation of populations of *Triatoma infestans* (Klug) (Hemiptera: Reduviidae). Bulletin of EntomologicalResearch 72: 617-629.

11. Rodríguez Morales, D. J. 1978. Historias de Vida como Adaptación a la Variabilidad Ambiental: Un Modelo de Simulación Aplicado a Triatominos. Tesis de Maestría. Instituto Venezolano de Investigaciones Científicas, Caracas 180 pp.

12. Da Rocha Carvalheiro, J. 1974. Tabuas de Vida e CapacidadeInata de Aumento Numérico de uma Populacao de *Triatoma infestans* em Condicoes de Laboratorio. III - Oviposicoes e Evolucao dos Ovos. Revista do Centro Academico Rocha Lima e do Hospital das Clinicas da Faculdade de Medicina de Riberao Preto da Universidad de Sao Paulo 6(1-2): 1-10.

13. Hack, W. H. 1955. Estudios sobre Biología del *Triatoma infestans* (Klug, 1834)(Hem., Reduviidae). Anales del Instituto de Medicina Regional (Universidad Nacional de Tucuman, Argentina, Publicación No 709) 4(2): 125-147.

**Table C.** Estimates of *T. infestans* net population growth rate (Ro) from the literature, under different laboratory conditions.

| Laboratory conditions | Ro estimate | Source |
| --- | --- | --- |
| Uncontrolled laboratory temperature with mean 24.3°C (± 3.26°C std. dev.); feeding source: hens; feeding frequency: twice a week | 162.3 | (1) |
| Controlled laboratory with constant temperature of 26°C; feeding source: hens; feeding frequency: once a week | 25.04 (± 19.80 std. dev.) | (2) |
| Controlled laboratory with constant temperature of 26°C; feeding source: hens; feeding frequency: once a week | 17.90 (17.74 - 18.07 95% CI) | (3) |
| Controlled laboratory with constant temperature of 26°C; feeding source: human blood in an artificial feeder; feeding frequency: once a week | 12.71 (12.56 - 12.87 95% CI) | (3) |
| Controlled laboratory with constant temperature of 26°C; feeding source: hens; feeding frequency: once a week | 18.6 (± 16.2std. dev.) | (4) |

**Bibliographic sources**:

1. Da Rocha Carvalheiro, J. 1974. Tabuas de Vida e Capacidade Inata de Aumento Numérico de uma Populacao de *Triatoma infestans* em Condicoes de Laboratorio. IV. Mortalidade e Crecimento da Populacao. Revista do Centro Academico Rocha Lima e do Hospital das Clinicas da Faculdade de Medicina de RiberaoPreto da Universidad de Sao Paulo 7(3-4): 65-85.
2. Rabinovich, J. E. 1972. Vital statistics of Triatominae (Hemiptera: Reduviidae) under laboratory conditions. I. *Triatoma infestans* Klug. Journal of Medical Entomology 9(4): 351-370.
3. Medone, P., A. Balsalobre, J. E. Rabinovich, G. A. Marti. 2015. Not only a question of feed or not to feed: demographic fitness of *Triatoma infestans* (Reduviidae: Hemiptera) fed on human blood. Submitted for publication to the Journal of Medical Entomology.
4. Rodríguez Morales, D. J. 1978. Historias de Vida como Adaptación a la Variabilidad Ambiental: Un Modelo de Simulación Aplicado a Triatominos. Tesis de Maestría. Instituto Venezolano de Investigaciones Científicas, Caracas 180 pp.
