# Supplementary material for: Biological Control of the Chagas Disease Vector Triatoma infestans with the Entomopathogenic Fungus Beauveria bassiana Combined with an Aggregation Cue: Field, Laboratory and Mathematical Modeling Assessment
Source: PLoS Negl Trop Dis. 2015 May 13;9(5):e0003778. doi: 10.1371/journal.pntd.0003778 (PMC4430541; doi:10.1371/journal.pntd.0003778)
Supplement: S3 File — (DOCX) [file pntd.0003778.s003.docx]

**S3 File**

**Field data and model comparison.**

**Table A.** Main differences between the model and the field conditions.

| **Characteristic/property** | **Field** | **Model** |
| --- | --- | --- |
| 1. Population numbers | Manual sampling recording, on average, a fraction between 5% and 25% of real population | Population is calculated total population |
| 2. Population sex | Both sexes | Only females |
| 3. Population structure | Adults andpooled nymphal stages | All stages separate |
| 4. Population initial conditions | Given by sample of November 2009, variable among houses | Unique population initial condition |
| 5. Nature of the data | Infected bugs result from a 60-day accumulation | Infected bugs are calculated daily |
| 6. Environmental climatic conditions | Population sampled in summer, with population “explosion” due to high temperatures | Assumes constant temperatures |
| 7. Environmental habitat conditions | Houses heterogeneous in number of people, animals, construction materials, etc. | Variations in habitat not represented |
| 8. Rooms and infective boxes | Number of rooms variable (and so number of boxes per house) | Only one fixed number of boxes per house |
| 9. Life history parameters (mainly development time and survival) | Varying along time and especially seasons | Constant during simulated time |
| 10. Biological control parameters (*α* and *Efic*) | Variable in different houses and with time for a given house | Constant during simulated time |
| 11. Dynamic processes | Density dependent population regulation/dispersal | No population regulation nor dispersal |
| 12. Resulting type of data | Two points in time: day 1 and day 60 | Any number of daily points |

Despite all these differences, we attempted the adaptation of the field data and the model in order to make both as comparable as possible. For this purpose we proceeded as follows:

1. We converted the sampled house number of bugs of our field data in Salta (collected with one man-hour/house effort) in total number of bugs basing the conversion on the proportion of triatomines that can be collected with that unit effort; Rabinovich et al. (1995) published the relationship between the one man-hour/house collection effort and total house population (from a census based on complete demolition) with the species *Rhodnius prolixus*; we used that information and, after weighting by the stage distribution (based upon the stage distribution estimated for *T. infestans* by Gurtler et al. 1992), we found that, on the average, 6.9% of the nymphs and 22.9 % of the adults infesting a house could be detected by a one man-hour/house collection effort; we couldn’t find a similar relationship for *T. infestans*, but Ronderos et al. (1981) provided a similar figure (7.9% for the overall population, i.e., nymphs and adults) for this species based upon a regression using the cumulative removal sampling method and full demolition of a house. Thus we proceeded to use the *R. prolixus* information of 6.9% and 22.9 % for the nymphs and adults, respectively. The results of this conversion are given in Table B.

**Table B**. Total number of *T. infestans* per house (per stage and total) estimated from the conversion of the one man-hour/house collection effort into total insect numbers using a conversion factor of 6.9% and 22.9 % for the nymphs and adults, respectively. N= nymphs, A= adults, T= total.

|  | Initial survey (November 2009) | | | Non-infected (January 2010) | | | Infected  (January 2010) | | |
| --- | --- | --- | --- | --- | --- | --- | --- | --- | --- |
| House | N | A | T | N | A | T | N | A | T |
| 1 | 319 | 48 | 367 | 1101 | 118 | 1219 | 101 | 17 | 118 |
| 2 | 275 | 22 | 297 | 0 | 13 | 13 | 87 | 35 | 122 |
| 3 | 420 | 148 | 569 | 855 | 61 | 916 | 43 | 9 | 52 |
| 4 | 246 | 39 | 286 | 377 | 61 | 438 | 14 | 4 | 18 |
| 5 | 43 | 31 | 74 | 232 | 31 | 262 | 0 | 9 | 9 |
| 6 | 116 | 57 | 173 | 362 | 87 | 450 | 14 | 31 | 45 |
| 7 | 159 | 44 | 203 | 232 | 9 | 241 | 0 | 4 | 4 |
| 8 | 522 | 9 | 530 | 72 | 9 | 81 | 203 | 61 | 264 |
| Average | 263 | 50 | 313 | 404 | 49 | 453 | 58 | 21 | 79 |
| Lower 95% CI | 133 | 15 | 173 | 92 | 16 | 13 | 1 | 5 | 8 |
| Upper 95% CI | 392 | 85 | 452 | 716 | 81 | 792 | 115 | 37 | 152 |

1. For the purpose of a population simulation with the mathematical model we had to provide the population initial conditions; as the field estimates pooled all nymphs in only one class, we had to separate that nymphal total into their respective stages; to that end we used the stage distribution estimated for *T. infestans* by Gurtler et al. (1992), after averaging for the three strata (roof, palm wall, and mud wall) of the houses of that study:

**Table C**. Stage distribution of a *T. infestans* domiciliary population as estimated by Gurtler et al. (1992), by house strata, and averaged for its use in the simulation model’s initial conditions.

| Stage | Stratum | | | Average  Density as a proportion |
| --- | --- | --- | --- | --- |
|  | Roof | Palm wall | Mud wall |  |
| I | 0.270 | 0.375 | 0.225 | 0.290 |
| III | 0.094 | 0.219 | 0.220 | 0.178 |
| III | 0.139 | 0.137 | 0.267 | 0.181 |
| IV | 0.096 | 0.063 | 0.114 | 0.091 |
| V | 0.142 | 0.084 | 0.079 | 0.102 |
| Adult | 0.258 | 0.122 | 0.095 | 0.158 |

Thus, the total nymph population estimated for the initial survey of November 2009 (see Table B) was affected by the proportion of each nymphal stage, and provided to the simulation model as the initial population condition. The initial number of eggs was never estimated, so we assigned to the egg stage class the initial number of 390 eggs based on the 313 adults estimated for November 2009, see Table B (=313 adults/2 to consider only females, times 0.5 eggs per female per day, times 10 days of egg-laying, which is the average development time of an egg).

1. As the matrix mathematical model is based on a female only population, all those initial conditions were halved, and the final results of the simulations were doubled to go back to results expressed as total population (both sexes); that is, for both procedures we are assuming a 50% sex-ratio of all stages (nymphs and adults) as a rough approximation.
2. To compare the model’s results with the field data we had to adapt the model to account for the total (accumulated) number of insects that died by fungus infection; this was necessary because the original model only showed the daily number of insects, both alive and dead by infection, while the “infected insects” of the field data represented the total number of insects found between the initial survey and the January 2010 survey.
3. We then used the simulation model assigning an uniform random value to some of the model parameters, which were the ones that assumed would be the most different in the field as compared to their estimation in the laboratory. We selected five parameters for this adaptation; three are the ones already described in the paper (Efficacy, a, and N), and we added two more parameters to adapt the possible effect of temperature, house construction material, number of rooms, etc. on development time and daily survival (which we called “Development time factor” and “Survival factor”), and were used as a multiplicative factor for the development time and the daily survival, respectively; these five parameters are identified in Table D, showing the limits assigned to them to extract a uniform random value (these limits were relatively arbitrary, but within the range of a plausible behavior).

**Table D**. Lower and upper limits used to apply the procedure of extracting a random number with a uniform distribution for five parameters of the simulation model.

| Parameter | Lower limit | Upper limit |
| --- | --- | --- |
| Efficacy | 0.001 | 0.5 |
| Fecundity | 0.3 | 1 |
| N (number of boxes) | 1 | 12 |
| Development time factor | 0.75 | 1 |
| Daily survival factor | 0.75 | 1 |

1. We then processed the model with our program “*Glimso*”, using the PSO tool (“Particle Swarm Optimization”) which is based on a procedure developed by Kenedy and Eberhart (1995) that optimizes the process of selecting a random value within the established limits, trying to minimize a cumulative sums of squares function (SSQ) between the simulated and observed values of the four variables of interest: infected and non-infected nymphs and adults, respectively (Table E).

**Table E.** Results of the “Particle Swarm Optimization” tool (last line, row labeled Model) and the field average values with their 95% confidence intervals.

|  | Non-Infected Nymphs | Non-Infected Adults | Infected Nymphs | Infected Adults | Total population |
| --- | --- | --- | --- | --- | --- |
| Field Average | 404 | 49 | 58 | 21 | 532 |
| Lower 95% CI | 92 | 16 | 1 | 5 | 114 |
| Upper 95% CI | 716 | 81 | 115 | 37 | 950 |
| Model (minimum SSQ) | 403 | 23 | 127 | 13 | 566 |

It can be seen that the best fitted values by the SSQ criterion conform well within the confidence intervals of the field values, with the exception of the infected nymphs, which remain slightly higher than the upper limit of the field value. The best fitted results of the last row of Table 4 corresponded with the following values of the parameters that were modified: Efficacy= 0.058, Fecundity= 0.56, N= 2, Development time factor= 0.85, and Daily survival factor= 0.82.

1. Once the best estimation of the five parameters were obtained, we proceeded to make a new simulation using those best fitted values as the mean of a normal distribution, and assigned an arbitrary coefficient of variation of 50% to the parameters’ distribution; we ran 100 simulations to obtain 100 values for each of the four variables of interest (infected and non-infected nymphs and adults).

**Bibliographic sources**:

1. Gürtler, R., M. C. Cecere, D. N. Rubel and N. J. Schweigmann. 1992. Determinants of the Domiciliary Density of *Triatoma infestans*, Vector of Chagas Disease. Medical and Veterinary Entomology 6: 75-83.
2. Kenedy, J. and R. Eberhart. 1995. Particle Swarm Optimization. In: Proceedings of IEEE International Conference on Neural Networks, Australia, vol. IV, pp. 1942–1948.
3. Rabinovich, J. E., R. E. Gürtler, J. A. Leal and D. Feliciangeli de Piñero. 1995. Density estimates of the domestic vector of Chagas' Disease, *Rhodnius prolixus* Stål (Hemiptera: Reduviidae), in rural houses in Venezuela. Bulletin of the World Health Organization 73(3): 347-357.
4. Ronderos, R. A., J. Schnack, J. Ghilini y G. Spinelli. 1981. Estudio Ecológico sobre una Población Domiciliaria de *Triatoma infestans* Klug de la Provincia Biogeográfica Chaqueña. ECOSUR 8(15): 1-24.
